# Comprehensive antifungal investigation of natural plant extracts against *Neosartorya* spp. (*Aspergillus* spp.) of agriculturally significant microbiological contaminants and shaping their metabolic profile

**DOI:** 10.1038/s41598-024-58791-4

**Published:** 2024-04-10

**Authors:** Wiktoria Maj, Giorgia Pertile, Sylwia Różalska, Kamil Skic, Magdalena Frąc

**Affiliations:** 1 Institute of Agrophysics, Polish Academy of Sciences, Doświadczalna 4, 20-290 Lublin, Poland; 2 Department of Industrial Microbiology and Biotechnology, Faculty of Biology and Environmental Protection, University of Łódź, Banacha Street 12/16, 90-237 Łódź, Poland

**Keywords:** Fungal biology, Fungal ecology, Food microbiology, Agroecology

## Abstract

Fungi belonging to the genus *Neosartorya* (teleomorph of *Aspergillus* spp.) are of great concern in the production and storage of berries and fruit-based products, mainly due to the production of thermoresistant ascospores that cause food spoilage and possible secretion of mycotoxins. We initially tested the antifungal effect of six natural extracts against 20 isolates of *Neosartorya* spp. using a traditional inhibition test on Petri dishes. Tested isolates did not respond uniformly, creating 5 groups of descending sensitivity. Ten isolates best representing of the established sensitivity clusters were chosen for further investigation using a Biolog™ MT2 microplate assay with the same 6 natural extracts. Additionally, to test for metabolic profile changes, we used a Biolog™ FF microplate assay after pre-incubation with marigold extract. All natural extracts had an inhibitory effect on *Neosartorya* spp. growth and impacted its metabolism. Lavender and tea tree oil extracts at a concentration of 1000 µg mL^−1^ presented the strongest antifungal effect during the inhibition test, however all extracts exhibited inhibitory properties at even the lowest dose (5 µg mL^−1^). The fungal stress response in the presence of marigold extract was characterized by a decrease of amino acids and carbohydrates consumption and an uptake of carboxylic acids on the FF microplates, where the 10 studied isolates also presented differences in their innate resilience, creating 3 distinctive sensitivity groups of high, average and low sensitivity. The results confirm that natural plant extracts and essential oils inhibit and alter the growth and metabolism of *Neosartorya* spp. suggesting a possible future use in sustainable agriculture as an alternative to chemical fungicides used in traditional crop protection.

## Introduction

In recent years, due to climate change, a continuous increase in temperature and a reduction in rainy periods can be observed, which has led to the frequent occurrence of poor-quality agricultural products. This combined with contamination by pathogenic and toxigenic fungi, is placing significant economic strain on agriculture, particularly for fruit and post-harvest products^1^. Approximately 25% of the global food supply is lost due to microbiological deterioration that occurred in the post-harvest phase. Furthermore, 92% of European and American juice producers reported having experienced mould or yeast contamination in the finished product and 89% of the producers reported having observed ingredient spoilage in general production^2^. Among fungi belonging to the group of microbiological food contaminants, the most important in the field of agriculture and food production are Heat Resistant Fungi (HRF). This group is heavily studied because of their ability to produce spores that allow them to resist temperatures above 75 °C, greatly diminishing the effectiveness of various pasteurization and sterilization techniques of fruit-derived food. HRF cause problems in the food chain connected to fruit (for example fruit juice and pulp)^3^. The most notorious fungi from the HRF group are *Byssochlamys*, *Eupernicillium*, *Hamigera*, *Neosartorya*, *Talaromycetes, Thermoascus, Rasamsonia*^4–6^. *Neosartorya* spp. (teleomorph of *Aspergillus* spp.) belong to section Fumigati and are one of the most recurring fungal threats in food production^7^. They are present worldwide and have been discovered in different environments: in soil^8^, sea water^9^, on common weeds^10^, grass^11^, wood^12^ and crops^13^. Additionally, *Neosartorya* spp. have been isolated from canned products^14^ and juices^15^ highlighting the importance of conducting studies on these fungi in order to find a natural method of controlling their spread in the agricultural filed. *Neosartorya* spp. are unique fungi, with additional defence mechanisms such as resilient cell walls containing substances such as trehalose, isobemisiose, neosartose and fischerose which enable them to survive drought and heat. In some cases, this genus is more resilient to stress than other genera in the HRF group, e.g. with *Neosartorya fischeri* outlasting *Talaromyces macrosporus* in drought conditions^16^. Many studies are being conducted on methods to block the propagation of spores, since this group of fungi are resistant to the high temperatures reached during pasteurization or sterilization and further increasing the temperature would lead to adverse effects on the quality of food through e.g. caramelization, evaporation and densification^17^. The importance of stopping the contamination not only concerns food spoilage, but also consumers’ health, as these potentially toxigenic fungi can produce mycotoxins such as fumitremorgin C, verruculogen and fischerin^18–20^. However, due to chemical fungicide overuse the European Union has launched the “Farm to Fork” strategy^21^. The need for alternative fungicide solutions stems from the need of protection of soil microbial diversity, soil quality and fertility, and groundwater quality. In accordance with this policy, alternative antimicrobial substances should be studied. One possible choice could be natural plant extracts, such as dry and oil extracts. Active ingredients of plant extracts can be removed from the whole plant or from parts of the plant (such as roots, stem, leaves, and flowers) due to use od different solvents, such as water, acetone, acetic acid, ethanol, ethyl acetate, methanol, and hexane^22^. The principal antifungal activity effect depends on the specific bioactive components such as alkaloids, phenols, flavonoids, terpenes, ketones, amines, and sulphides^23^.

In the field of microbiology, extensive research has been conducted on the antifungal properties of essential oils and plant extracts. Abdolahi et al.^24^ analysed the antifungal effect of different essential oils from ajowan, fennel, caraway against *Penicillium digitatum* and *Alternaria alternate*. Other studies identified antifungal effects of eugenol, thymol, and summer savory against the pathogenic fungus *Botrytis cinerea*^25–28^. Tripathi et al.^29^ reported a 100% inhibitory effect of various essential oils to *Botrytis cinerea*. Zabka et al.^30^ reported the effect of 14 plant extracts on different pathogenic fungi, including *Fusarium oxysporum*, *F. verticillioides*, *Penicillium expansum*, *P. brevicompactum*, *Aspergillus flavus*, and *A. fumigatus*. However, little research has been conducted on the antifungal effects of dry plant extracts and essential oils on heat resistant fungi. This could be particularly valuable considering the known antifungal properties of essential oils and plant extracts, and because these natural substances are non-toxic, biodegradable, environmentally non-persistent and declared as “Generally Regarded as Safe” (GRAS) and can be used in food production^22,23^.

Considering the economic losses and possible health hazard caused by highly resilient *Neosartorya* sp., it is necessary to search for additional methods of fungal control while simultaneously adhering to the new novel ecological directives. The main objective of this research was to compare sensitivity among *Neosartorya* spp. isolates to plant extracts, taking into the account possible differences between individual isolates. Furthermore, the antifungal properties of natural extracts were assessed. Additionally, metabolic profiling of selected isolates was performed to observe possible changes in metabolic capability of isolates varying in sensitivity. Morphological features of *Neosartorya* spp. were also visualized to confirm whether isolates of *Neosartorya* sp. presented pre-existing morphological features that could potentially influence their response towards antifungal plant extracts.

## Materials and methods

### Natural extracts

The antifungal effects of dry extracts (marigold and lavender) and essential oils (lavender, rosemary, clove, and tea tree) against *Neosartorya* spp. were analysed. Dry *Lavandula angustifolia* flower extract was produced by Greenvit Sp. z o.o. (Zambrów, Poland) using a maltodextrin carrier. *Calendula officinalis* dry extract was produced by Zrób Sobie Krem Kosmetyki Naturalne (Prochowice, Poland). All dry extracts were determined to be free from *Salmonella* spp. and *Escherichia coli*. Oil extracts were produced by Dr Beta^®^, FSZ Pollena-Aroma Sp. z o.o., (Nowy Dwór Mazowiecki, Poland) and registered in the Cosmetic Products Notification Portal (CPNP). Studied oils were as follows: tea tree oil (*Melaleuca alternifolia* leaf oil, limonene), eucalyptus oil (*Eucalyptus globulus* leaf oil, limonene), rosemary oil (*Rosmarinus officinalis* leaf oil, limonene, linalool, geraniol, eugenol), clove oil (*Eugenia caryophyllus* bud oil, eugenol, isoeugenol), lavender oil (*Lavandula angustifolia* oil, linalool, geraniol, limonene, citronellol).

The chemical nature of natural extracts makes them highly antimicrobial, thus rigorous material sterilization processes are not needed. To prepare stock solutions, each of the powdered plant extracts was resuspended and dissolved in sterile water to achieve a concentration of 1000 µg mL^−1^ and filtered via 0.22 µm filters (Millex^®^, Ireland). Natural oils were weighed and added to sterile water to establish the percent weight by volume (% w/v) of 1000 µg mL^−1^, thoroughly mixed by vigorous shaken until a cloudy emulsion without visible phase separation was formed and then filtered via 0.22 µm filters (Millex^®^, Ireland). All stock solutions were diluted to the following concentrations: 250, 150, 100, 50 and 5 µg mL^−1^. This method of oils preparation was used to not disturb naturally occurring reactions of volatile organic compounds (VOCs) found in essential oils during the incubation phase as e.g. ethanol and methanol are linked with fast VOCs dispersion^31^. Naturally, VOCs can disperse through water matrices while maintaining lasting effects on the environment^32^. Incubating fungi on moist media in contact with natural oils within sealed petri plates could potentially replicate the controlled atmosphere of transport or storage conditions of food as with time volatile compounds diffuse into air^33^. A water-based oil dispersion method was also chosen on the basis that common oil solvents such as ethanol or dimethyl sulfoxide (DMSO) may not be adequate in fruit production since side-effects such as altered ripening time^34^ or nutrient (e.g. carotenoids) synthesis disruption can occur due to their involvement^35^.

### Fungal isolates identification

*Neosartorya* spp. isolates were obtained from the Laboratory of Molecular and Environmental Microbiology, Institute of Agrophysics of Polish Academy of Sciences (Lublin, Poland). A total of 20 distinct isolates were selected by the sequencing Internal Transcribed Spacer region 1 (ITS1)^36^, and a fragment of the functional β-tubulin gene specific to this genus^4^. The N2F/2R and Af1F/1R primers have proven efficacy in distinguishing between *Neosartorya* spp. and *Aspergillus fumigatus*^4^. Accurate discrimination between *A. fumigatus* and other *Neosartorya* species is of utmost importance in the food industry and in this study because although they are morphologically similar, *A. fumigatus* is not well documented as a spoilage agent in heat-processed food products, contrary to *Neosartorya* genera well-known for food spoilage capabilities. Only isolates presenting PCR product bands for N2F/2R and not for Af1F/1R primers were utilized in this study to ensure that the samples were all withing the *Neosartorya* genus. This was further proven by high percent identity scores via BLAST+^37^. To facilitate comparison between the origin of the isolates, 8 isolates originating from soil and 12 from strawberry fruit were chosen. Prior to genetic identification, the isolates were cultivated on Potato Dextrose Agar medium (PDA, BioMaxima S. A., Lublin, Poland) at 30 °C. After 5 days of incubation, the mycelium was transferred into 2 mL tubes containing glass beads (250 mg 1.45 mm⌀ and 500 mg 3.15 mm⌀) and Lyse F buffer (EURx^®^, Poland) and later homogenized using FastPrep-24 homogenizer (MP Bio, United States) for 20 s at 4 m s^−1^. The DNA was extracted using the EURx GeneMATRIX Plant and Fungi DNA Purification Kit (EURx^®^, Poland)^38^. Three different PCRs were prepared for following genes: ITS1 (primers ITS1/ITS2^39^), β-tubulin gene (primers specific for *Neosartorya* spp. N2F/N2R and *Aspergillus fumigatus* Af1F/R^4^). PCRs were conducted using REDTaq^®^ ReadyMix™ (Sigma-Aldrich Co. LLC, USA), 10 μM of each primer and 2 μl of diluted fungal DNA. The amplification conditions were reported in Supplementary Table S1.

Each PCR product was analysed on a 2% agarose gel. PCR products were sequenced using Sanger sequencing method. The amplicons were purified using Exo-BAP mix (EURx, Gdańsk, Poland) and re-amplified with Big Dye^®^ Terminator v1.1 Reaction Mix (Thermo Fisher Scientific, Waltham, MA, USA). All samples were cleaned through the Performa^®^ DTR Cartridges (Edge BioSystems, Gainthersburg, MD, USA). Purified products were incubated at 95 °C for 180 s followed by an incubation at 4 °C for 180 s, and loaded into the Applied Biosystems 3130 Genetic Analyzer (Applied Biosystems, Foster City, CA, USA). Phylogenetic trees were obtained using MEGA 11 software^40^. In this analysis, some sequences of other HRF genera were added for comparison, all obtained from the National Centre for Biotechnology Information database (NCBI; http://www.ncbi.nlm.nih.gov). Additional internal transcribed spacer region sequences included: *Eupenicillium meridianum* (AM992114.1), *Hamigera insecticola* (NR137684.1), *Paecilomyces variotii* (NR130679.1), *Byssochlamys lagunculariae* (FJ389944.1), *Aspergillus nidulans* (NR133684.1), *Aspergillus niger* (NR111348.1), *Aspergillus flavus* (NR111041.1), *Neosartorya fischeri* (KF640700.1), *Neosartorya spinosa* (JN943589.1), *Neosartorya glabra* (JN943577.1), *Talaromyces udagawae* (MH860584.1), *Aspergillus* sp. (ON920551.1), *Neosartorya* sp. (MH472615.1) *Neosartorya* sp. (2) (MF681541.1), *Neosartorya laciniosa* (JN943581.1), *Neosartorya assulata* (HF545007.1). Additional beta tubulin sequences included: *Neosartorya glabra* (AF057323.1), *Neosartorya spinosa* (AF057329.1), *Neosartorya fischeri* (AF057322.1), *Talaromyces udagawae* (OK338783.1), *Aspergillus fumigatus* (OP646312.1), *Aspergillus* sp. (OL792696.1), *Neosartorya* sp. (KT253238.1), *Neosartorya* sp. (2) (EU220283.1), *Neosartorya laciniosa* (JX845620.1), *Neosartorya* sp. (3) (DQ114123.1), *Penicillium meridianum* (GU981660.1), *Hamigera insecticola* (LC076663.1), *Paecilomyces variotii* (MN153297.1), *Byssochlamys lagunculariae* (AY753353.1), *Aspergillus nidulans* (MK749993.1), *Aspergillus niger* (LC774552.1) and *Aspergillus flavus* (AF036803.1). Maximum likelihood tree illustrating the ITS1 phylogenesis was created using Tamura 3-parameter model with discrete gamma distribution, whereas the β-tubulin phylogenesis was created using Kimura 2-parameter model with discrete gamma distribution.

### Disc diffusion assay for testing fungal isolates sensitivity to plant extracts

The disc-diffusion inhibition test was used to determine an inhibitory effect of a specific plant extract on the growth of 20 *Neosartorya* spp. isolates. For this analysis several extracts were used, such as: lavender flower dry extract, pot marigold flower dry extract, lavender oil, tea tree oil, clove oil and rosemary oil. All natural substances were tested at concentrations of 1000 µg mL^−1^, 250 µg mL^−1^, 150 µg mL^−1^, 100 µg mL^−1^, 50 µg mL^−1^ and 5 µg mL^−1^. To test the effect of the inhibitory properties of these extracts on growth, Petri plates with PDA medium (BioMaxima S.A., Lublin, Poland) were inoculated with 300 µL of a *Neosartorya* spp. water suspension, as described in Pertile et al.^41^. To ensure a uniform number of cells inside the inoculum, fungal material included ascospores with mycelium collected from the previous 10 days old culture was placed in FF inoculating fluid (Biolog™, Hayward, CA, USA) to achieve 75% transmittance equating to approximately 4.44 × 10^4^ ascospores mL^−1^ (Supplementary Table S3). Moreover, to confirm the inoculum uniformity, the ascospores were counted in a cell counting chamber acc. to Thoma (Hirschmann Laborgeräte GmbH & Co. KG, Eberstadt, Germany). Taking into account that *Neosartorya* spp. fungi are heat resistant due to their ability to form resilient sexual spores, fungal growth with present ascospores was obtained. This was confirmed by SEM (Phenom ProX, Thermo Fisher Scientific, Waltham, MA, USA) (Fig. S5). Sterile paper discs (Whatman No. 1, 5 mm⌀) were placed on top of the medium and 30 µL of plant extract at each of the above concentrations was applied to each disc. Each combination of isolate, extract type and concentration was replicated three times. Three discs with the same concentration were placed in the same Petri dish. Petri plates were incubated at 30 °C for 10 days and the areas of growth inhibition were measured daily using a digital caliper.

### Sensitivity of *Neosartorya* spp. isolates to different concentrations of natural plant extracts using MT2 microplates

To assess the resource utilization, sensitivity, or resistance of *Neosartorya* spp. to the presence of analysed natural plant extracts, MT2 microplate assays were conducted (Biolog™, Hayward, CA, USA) following the methods described in Frąc et al.^42^. In accordance with the results from the inhibition test, multiple compounds and dilutions with the best antifungal properties were chosen. Selected natural extracts were marigold dry extract, lavender flower dry extract, lavender oil, rosemary oil, tea tree oil, and clove oil. Based on the previous results, each natural extract was tested at the following concentrations: 1000 µg mL^−1^, 150 µg mL^−1^, 50 µg mL^−1^ and 5 µg mL^−1^. Furthermore, on the basis of inhibition tests, the 20 analysed isolates were divided into 5 different groups according to their sensitivity to tested substances, from which 2 isolates per group were chosen to be studied via the MT2 analysis (Biolog™, Hayward, CA, USA).

Pure cultures of 10 fungal isolates were grown on PDA medium (BioMaxima S.A., Lublin, Poland) at 30 °C for 10 days. After the incubation period, ascospores with mycelium were collected and placed in a sterile filter bag with FF inoculating fluid (FF IF, Biolog™, Hayward, CA, USA) to perform homogenization. Subsequently transmittance of 75% equating to approximately 4.44 × 10^4^ ascospores mL^−1^ was achieved (Supplementary Table S3). Each Biolog™ microplate well was filled with 100 µL of natural extract and 50 µL of fungal inoculum. To allow a comparison of fungal development with and without the presence of natural extracts, a control was prepared which composed of fungal ascospores with mycelium suspended in 50 µL FF IF and mixed with 100 µL of water. Plates were incubated at 30 °C and analysed every 24 h for 10 days at the optical density (OD) of 490 nm (utilisation of substances) and 750 nm (fungal biomass production) using the Biolog™ MicroStation (Biolog™, Hayward, CA, USA). For each wavelength separately, the obtained results were corrected by subtracting values of background obtained for each the used dilution with each plant extracts, as they could interfere spectrophotometrical measurements and results. The blank samples were formed by extracts dilutions (100 µL) mixed with inoculating fluid solution (FF IF 50 µL) instead of inoculum in order to avoid background interference in this study. Then subcontracted values were used to calculate the Average Fungal Respiration Intensity (AFRI; for the optical density at 490 nm) and Average Fungal Growth Intensity (AFGI; for the optical density at 750 nm). Results of samples treated with natural extracts were then compared to the results attained by the fungi cultivated in control conditions without the contact with any natural extracts.

### *Neosartorya* spp. metabolic profile analysis—following pre-incubation with marigold extract

To observe changes in the metabolism of *Neosartorya* spp. after pre-incubation in the presence of marigold extract, the FF microplate (Biolog™, Hayward, CA, USA) assay was carried out^43–45^. For this experiment, only marigold extract was selected as its low concentration permitted mycelial growth and accession of satisfactory quantity of fungal material. For the control group, pure cultures of fungi were grown on PDA medium (BioMaxima S. A., Lublin, Poland) at 30 °C for 10 days; whereas the test group was grown under the same conditions with an extra addition of marigold extract at a concentration of 150 µg mL^−1^ on PDA media. Each treatment was prepared in triplicate. Ascospores with mycelium were collected from the surface of each plate and placed in a sterile filtering bag with FF inoculating fluid (Biolog™, Hayward, CA, USA) with the subsequent adjustment of transmittance to 75%. FF plates were inoculated with 100 µL of fungal suspensions and incubated at 30 °C for 10 days. Optical density was measured every 24 h at 490 nm and 750 nm. Carbon sources in the FF plate can be divided into 6 groups (Supplementary Fig. S1): amines and amides, carbohydrates, polymers, amino acids, carboxylic acids and miscellaneous^46,47^. The number of different substrates used by individual isolates was used to determine functional diversity, which was expressed as substrate richness (R). The diversity amongst the isolates was determined by the utilization of substrates and expressed as the Average Fungal Respiration Intensity (AFRI; for the optical density at 490 nm) and Average Fungal Growth Intensity (AFGI; for the optical density at 750 nm). Within both the test and control groups at each measured wavelength, the optical densities of each individual well were subsequently adjusted by subtracting the optical density of the water control (inoculum resuspended in water) at the corresponding measurement hour.

### Scanning electron and fluorometric microscopy analyses

Five isolates were selected based on difference in their sensitivity levels to natural extracts were cultivated for 10 days on PDA medium (BioMaxima S. A., Lublin, Poland) and observed using an electron microscope, Phenom ProX (Thermo Fisher Scientific, Waltham, MA, USA). Samples were mounted on aluminium supports using conductive double-sided carbon adhesive tape, dried under silica gel and coated with a 5 nm layer of gold (sputter coater, CCU-010 LV, Safematic GmbH, Zizers, Switzerland) prior to SEM analysis. The pictures were captured using a BS detector at an accelerating voltage of 10 kV, at 200× magnification and 5000× magnification.

Three of these five isolates were selected for further examination under a ZEISS LSM 510 Meta Confocal Microscope. After 5 days of cultivation on PDA medium approximately half of the ascospores with mycelium was harvested and visualized without fluorescence (light microscopy). The remaining fungal material was added to a wash buffer (2% d-glucose and Na-HEPES) and subsequently centrifuged at 10,000×*g* for 5 min. After the removal of the supernatant, the pellet was re-suspended in 1 mL of wash buffer and 1 µL of FUN 1 cell stain (10 mM solution in DMSO; Thermo Fisher Scientific Corporation, USA) was added. Samples were incubated at 30 °C in the dark for 5 min. Finally, the stained suspension was transferred onto a glass slide and covered with a coverslip. Cells were imaged with filters: excitation 488 nm, emission 530 nm. In parallel, photographs of the tested fungal isolates were taken under unfiltered light of to visualize possible differences in the production of spores.

### Statistical analysis

For the results from the inhibition tests, heatmaps with cluster analysis were calculated through Euclidian distance and Wards’ method respectively and prepared with R software v 4.3.1 (packages: *ComplexHeatmap*, *circlize*). Statistical tests were used to determine the differences between the isolates, effects of natural extracts and their concentrations on creating inhibition zones or absorbance values (MT2, FF). The initial phase involved assessing dataset normality using the Shapiro–Wilk test and examining variance homogeneity using the Levene test^48^. Since the datasets did not meet the criteria for parametric tests, the results were analysed by performing Kruskal–Wallis test and post hoc Wilcoxon Mann Whitney test using TIBCO Statistica^®^ software (Version 13.3). To visualize microplate tests results, we created heatmaps and bar graphs to demonstrate the similarities and differences in carbon utilization patterns among the different isolates. Data is presented at 95% confidence intervals with statistical significance at p < 0.05. Additional analyses based on OD readings included Substrate Richness (R index), Average Fungal Respiration Intensity (AFRI) and Average Fungal Growth Intensity (AFGI) to assess the functional diversity, impact of incubation time, isolate and carbon source type as in Oszust et al.^43^, Frąc et al.^49^. To further explore the carbon utilization a cluster analysis was performed, using a dendrogram created through the Ward method and Sneath's dissimilarity criterion at 66% and 33% (TIBCO Statistica^®^ software (Version 13.3).

## Results

### Fungal isolates identification

The identity of isolates was initially determined using the specific PCR’s and then confirmed by sequencing. Obtained products for the ITS1 gene were approximatively 240 bps (Supplementary Fig. S2A) and enabled an approximate placement of isolates at *Neosartorya*/*Aspergillus* genus level. The isolates presented a PCR-product for the N2F/N2R primers (Supplementary Fig. S2B) while they did not present any PCR product for *A. fumigatus* primers (primer Af1F/Af1R; Supplementary Fig. S2C) which effectively excluded them from relation to *A. fumigatus* species. The isolates were identified to genus level as *Neosartorya* spp. based on the Internal Transcribed Spacer (ITS) and β-tubulin sequences during Sanger sequencing. All obtained sequences were deposed in the National Centre for Biotechnology Information (NCBI; http://www.ncbi.nlm.nih.gov; see Supplementary Table S2).

**Figure 1:**
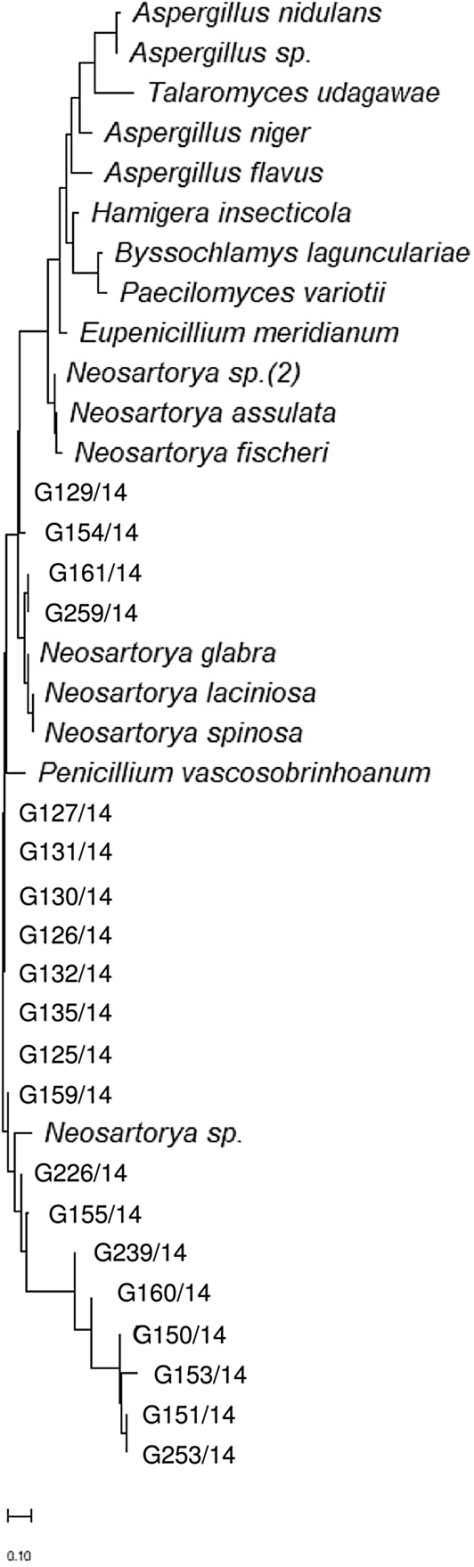
Phylogenetic tree based on ITS1 sequences of isolates used in this study and other published fungal sequences.

**Figure 2:**
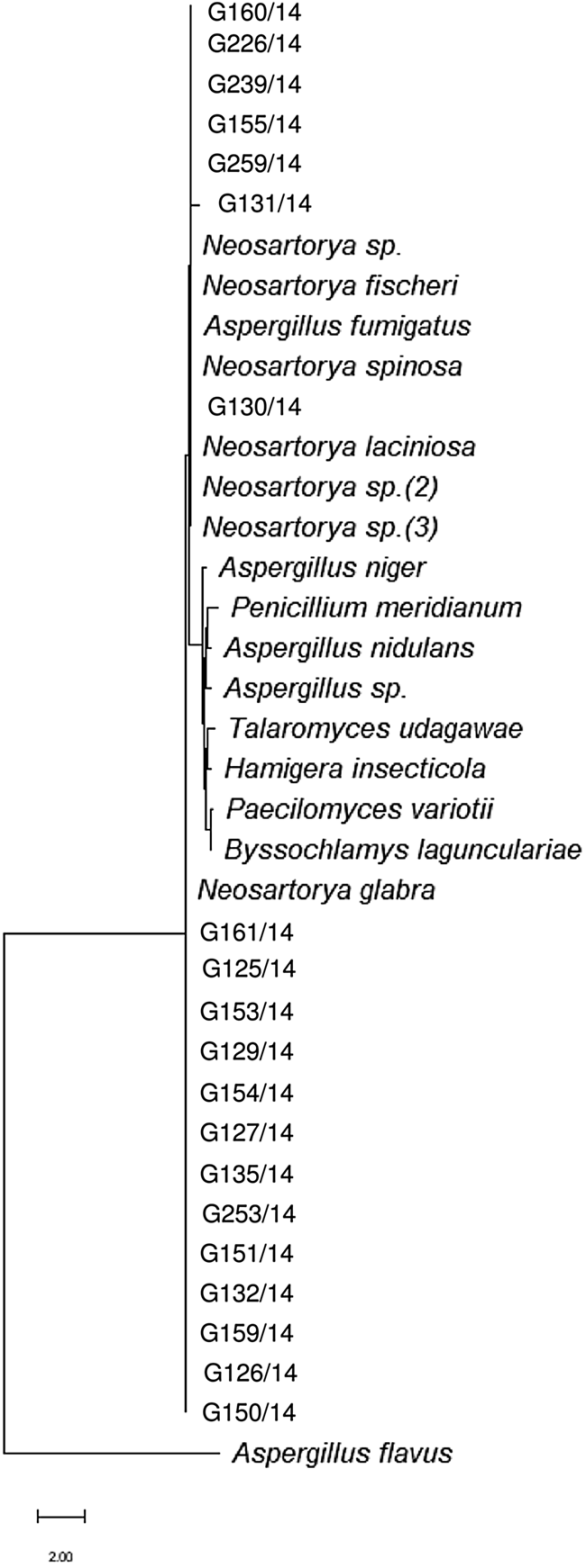
Phylogenetic tree based on β-tubulin gene sequences of isolates used in this study and other published fungal sequences.

After analysing the sequences obtained through specific phylogenetic trees (Figs. 1, 2) it was observed that the ITS1 gene sequences of the isolates were grouped closely with *Aspergillus* spp. and *Neosartorya* spp. creating a separate cluster from other HRF (*Eupenicillium meridianum, Hamigera insecticola, Paecilomyces variotii, Byssochlamys lagunculariae*, *Talaromycetes udagawe*; Fig. 1). Sequences obtained for the functional β-tubulin gene confirmed that the isolates belonged to the genus *Neosartorya* creating a cluster completely separated from *Aspergillus* spp. and other fungi (Fig. 2).

### Sensitivity analysis of *Neosartorya* spp. isolates to plant extracts

**Figure 3:**
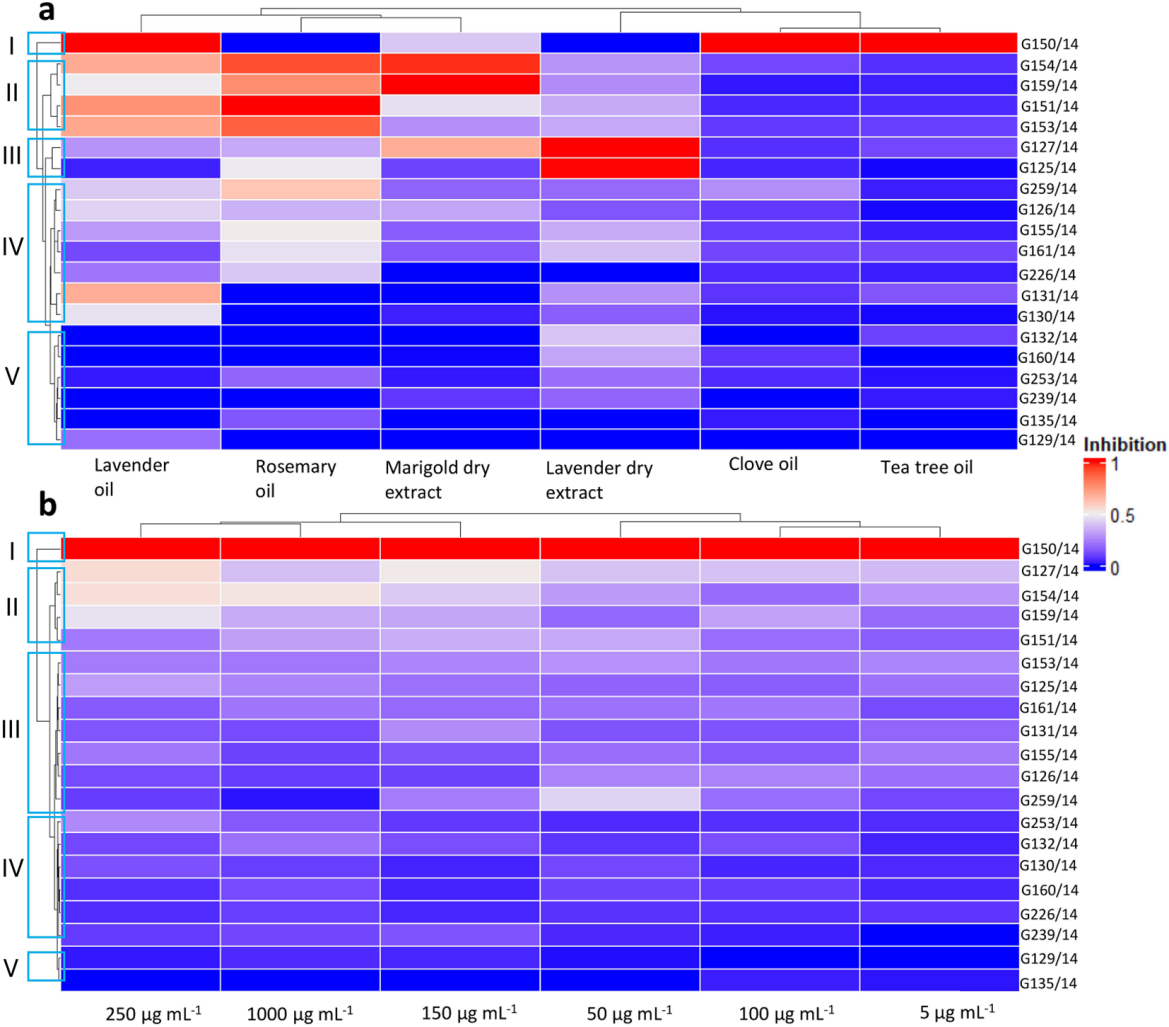
Heatmaps presenting the inhibition profile of different *Neosartorya* spp. isolates. Pooled results are shown for all concentrations of natural substances (**a**) and concentrations for all natural substances (**b**). Blue rectangles on the right signify isolate clusters.

The plant extracts (dry lavender flower, dry marigold, as well as lavender, tea tree, clove and rosemary oils) were effective at inhibiting *Neosartorya* spp. during the inhibition tests (Figs. S3 and S4). Essential oils had a stronger inhibitory effect than plant extracts, with rosemary and lavender oils the most potent (Fig. 3A). Clove and tea tree oils inhibited the growth of *Neosartorya* spp. to a lesser degree as the two other oils. As illuminated by results focused on the concentration, the medium dose (250 µg mL^−1^) of natural extracts tended to yield the strongest antifungal effect against all the tested isolates of *Neosartorya* spp. (Fig. 3B).

Based on cluster analysis, the 20 tested isolates were divided into 6 groups according to their susceptibility to the plant extracts. As observed in Fig. 3A, the isolates could be divided as follows: the most sensitive isolate G150/14 and the sensitive group with isolates G154/14, G159/14 and G151/14; a slightly higher than average sensitivity for G127/14 and G125/14; an average sensitivity for the group with G259/14, G126/14, G155/14, G161/14, G226/14, G131/14 and G130/14; and the lowest sensitivity group containing G132/14, G160/14, G253/14, G239/14, G135/14, and G129/14. Figure 3A,B showed that isolate G150/14 presented a different degree of sensitivity when compared to the other isolates of the *Neosartorya* genus. This preliminary analysis, aside from guiding the decision on which natural extracts and concentrations exhibited the best antifungal effect, also underscored a non-uniform response among the 20 tested fungal isolates, indicating a potential intraspecific difference.

**Figure 4:**
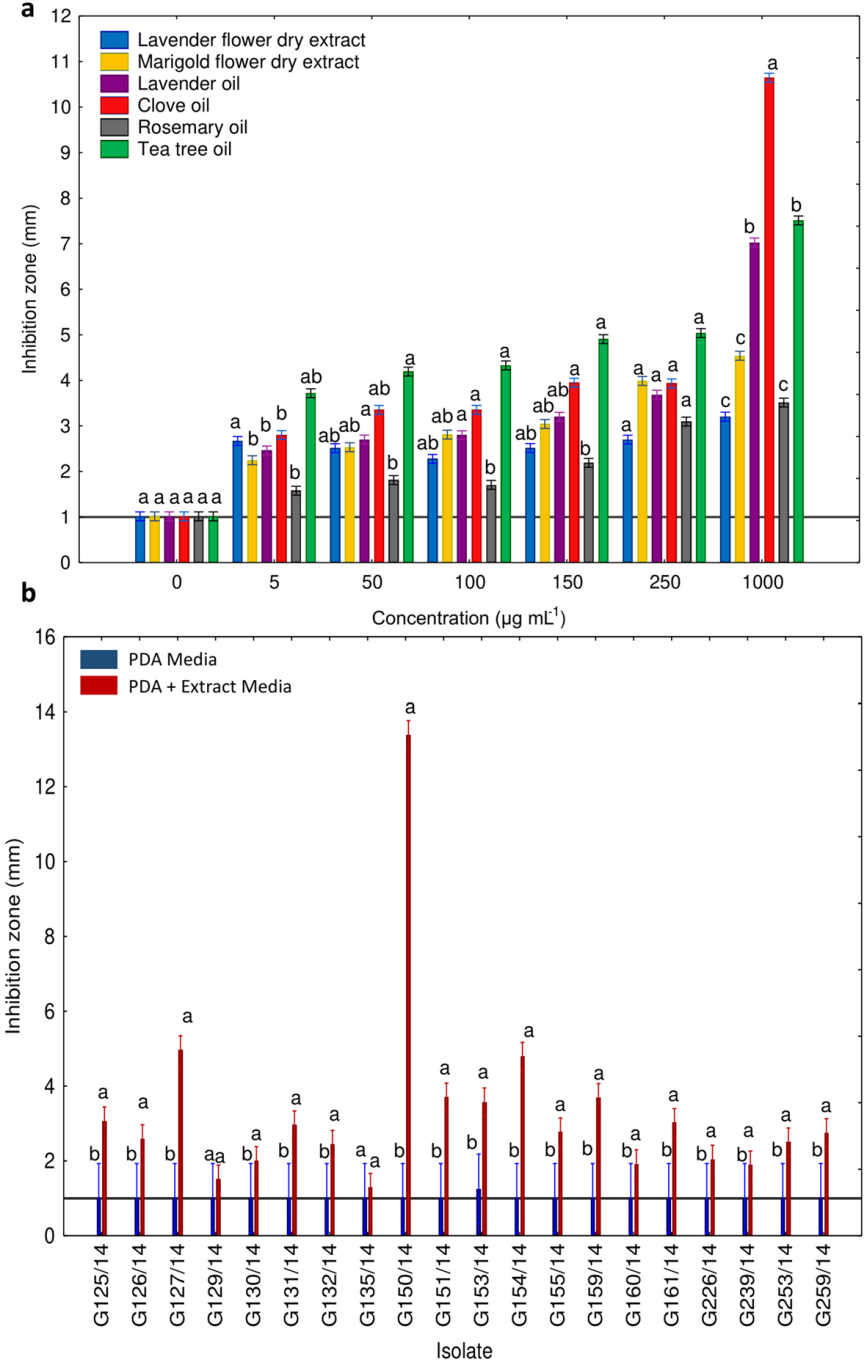
*Neosartorya* spp. isolates' sensitivity to natural extracts during disc diffusion inhibition test. Insights from analysed concentrations (**a**) and individual isolate responses to natural extracts (**b**). Significant differences indicated by letters (Wilcoxon test, p < 0.05).

The results were also analysed for of both the extract type and concentration together (Fig. 4A). The concentration of 1000 µg mL^−1^ seemed to be the most effective for all extracts in inhibiting *Neosartorya* spp., with clove oil at 1000 µg mL^−1^ presenting the highest antifungal effect on tested isolates among all analysed natural extracts. Interestingly, lavender flower dry extract presented a more or less uniform inhibitory ability regardless of dosage (ranging from a maximum of 1000 µg mL^−1^ and minimum of 100 µg mL^−1^). Marigold dry extract, lavender oil, clove oil, tea tree oil and rosemary oil presented an increasing trend of inhibition with increasing dosage. However, the antifungal capabilities did not increase in a linear manner for clove oil, lavender dry extract and rosemary oil. The results indicated that tea tree oil exhibited the highest inhibition towards *Neosartorya* spp. in all of the analysed concentrations (with the exception of 1000 and 5 µg mL^−1^).

The inhibition zones for each isolate were later compared with their respective controls (Fig. 4B). A large difference between the control and the group of isolate G150/14 was immediately evident, thus confirming the results in Fig. 3A,B and deeming the isolate the most sensitive towards contact with the plant extracts. The other isolates were categorized into groups based on the recorded inhibition zones: those with an high inhibition area (including G127/14, G151/14, G154/14, and G159/14), and those with an average inhibition zone (comprising of G153/14, G125/14, G126/14, G131/14, G155/14, G161/14 and G259/14) and low inhibition zone (G130/14, G132/14, G160/14, G226/14, G239/14, G253/14). Two isolates (G129/14 and G135/14) showed no growth difference and can therefore be described as a distinct group displaying the highest resistance to incubation with the plant extracts.

Ten strains were selected on the basis of the results from the inhibition test and further investigated on MT2 and FF microplates. Taking into account all results gathered thus far, 5 groups could be distinguished in terms of sensitivity (from the most to the least sensitive): G150/14 and G127/14 (I group); G226/14 and G132/14 (II group); G154/14 and G151/14 (III group); G130/14 and G129/14 (IV group); G135/14 and G160/14 (V group).

### Fungal sensitivity assay during the exposure to different concentrations of essential oils and natural extracts in MT2 microplates

**Figure 5:**
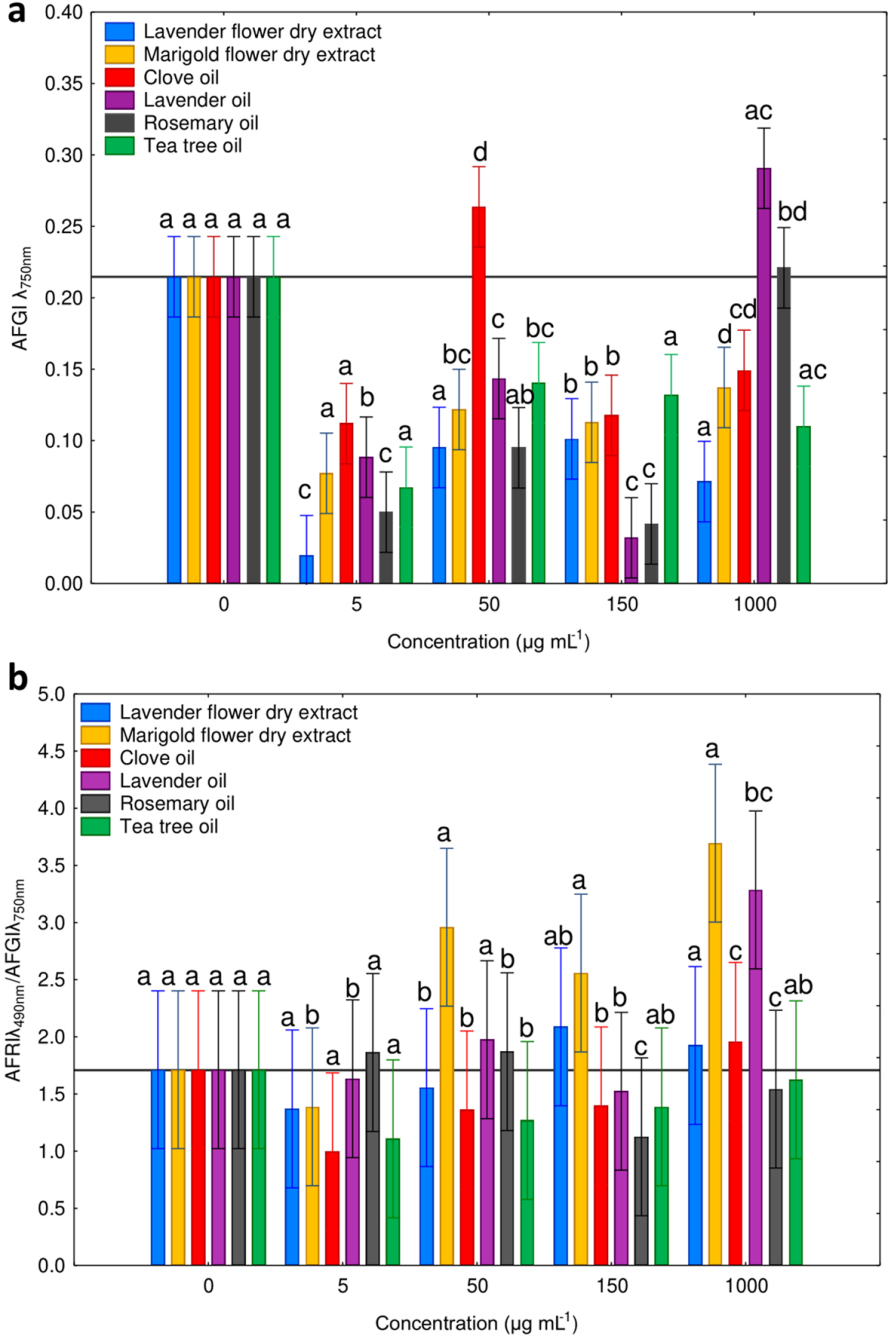
*Neosartorya* spp. isolates’ sensitivity to natural extracts during MT2 microplate assay. AFGI (Average Fungal Growth Intensity) for different treatments and their concentrations (**a**); Ratio of AFRI (Average Fungal Respiration Intensity)/AFGI for different treatments and their concentrations (**b**). Letters indicate significant differences (Wilcoxon test, p < 0.05).

The combined data from all 20 isolates was analysed. All analysed substances showed an inhibition towards the AFGI in the MT2 microplates, with the exception of lavender and rosemary oils at 1000 µg mL^−1^ and clove oil at 50 µg mL^−1^, which did not present an effective inhibition on the biomass production (Fig. 5A). The rest of the studied substance decreased the growth ability of *Neosartorya* spp., with the most effective substances being lavender flower dry extract (5 µg mL^−1^), and lavender and rosemary oils (150 µg mL^−1^). Rosemary and lavender oils showed an inhibitory effect at 5, 50, and 150 µg mL^−1^ and clove oil presented an inhibitory effect at: 5, 150, and 1000 µg mL^−1^. Tea tree oil was the most effective at a concentration of 5 µg mL^−1^, the same concentration at which marigold dry extract and clove oil exhibited the highest antifungal properties.

A low ratio between the AFRI and AFGI indicates a more efficient metabolism, due to a relatively large fungal biomass production despite low uptake of available substrates in plate wells. In contrast, a higher ratio indicates a smaller biomass (as indicated by low OD values at 750 nm) but with high respiration rates (as indicated by high OD values at 490 nm). This indicates a state of heightened metabolic activity or stress, where the fungal organism is experiencing a challenging metabolic situation^47,50^. The ratio between AFRI/AFGI differed for all tested concentrations of extracts and was different from the control (Fig. 5B). Higher ratio values mark fungal substrate stress which was especially prominent when dry marigold flower extract was used (1000 µg mL^−1^, 150 µg mL^−1^, 50 µg mL^−1^). Substrate related stress was also detectable when other substances were used: lavender oil (1000 µg mL^−1^ and 50 µg mL^−1^), rosemary oil (50 µg mL^−1^ and 5 µg mL^−1^), lavender flower dry extract (1000 µg mL^−1^ and 150 µg mL^−1^), and clove oil (1000 µg mL^−1^). Only rosemary oil could induce metabolic stress at a concentration of 5 µg mL^−1^ and promote better metabolism than the control when used at the highest concentration (1000 µg mL^−1^). Tea tree oil did not cause metabolic stress in the isolates during the MT2 microplate assay.

**Figure 6:**
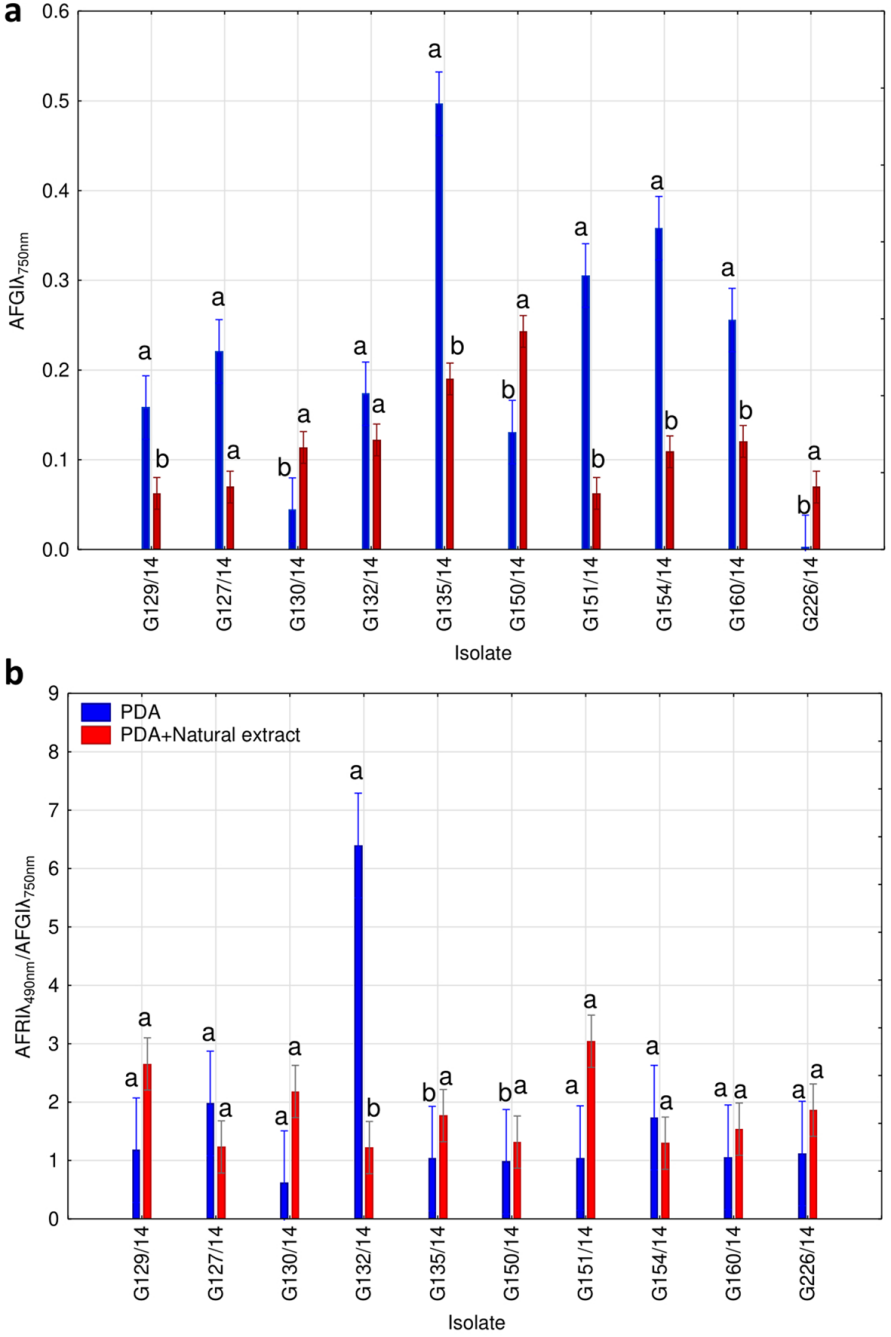
Evaluation of the *Neosartorya* spp. isolates’ sensitivity to natural extracts using a MT2 microplate assay. AFGI (Average Fungal Growth Intensity) of analysed isolates—control and test group (**a**); AFRI (Average Fungal Respiration Intensity)/AFGI of analysed isolates—control and test group (**b**). Different letters indicate significant differences (Wilcoxon test, p < 0.05).

The data from the MT2 assay was also analysed for all of the isolates separately, taking into account values obtained from the control and the group influenced by natural extracts (Fig. 6). After examining the AFGI index (Fig. 6A), it becomes evident that each group of isolates exhibited a distinct behaviour when exposed to these natural extracts. One group of isolates (G129/14, G127/14, G135/14, G151/14, G154/14, and G160/14) displayed a reduction in fungal biomass production upon exposure to natural extracts. Conversely, only three isolates (G130/14, G150/14, and G226/14) exhibited an increase in biomass production following exposure to natural substances. Isolate G132/14 showed no difference between the control and natural treatments. From the perspective of the AFRI/AFGI ratio, it was observed that only a few isolates displayed significant differences between the two treatments (Fig. 6B). Specifically, one isolate (G132/14) demonstrated a significantly lower value of AFRI/AFGI ratio after exposure to natural extracts compared to the control, indicating a higher metabolic efficiency and decrease in fungal biomass production, while isolate G135/14 exhibited a higher value of AFRI/AFGI ratio indicating a lower metabolic efficiency and an increase in biomass when exposed to the natural extracts.

**Figure 7:**
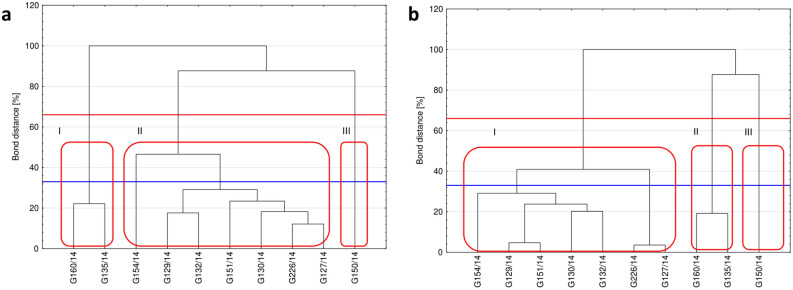
Cluster analysis of *Neosartorya* spp. isolates. Clusters according to the Average Fungal Respiration Intensity (**a**) and Average Fungal Growth Intensity (**b**) with the lines representing the Sneath criterion at 33% (blue line) and at 66% (red line) and numeration I, II, III for observable clusters.

The effect of fungal growth in response to the natural extracts was examined using the MT2 microplate. Slight variations in metabolic profiles were observed among the fungal isolates. Four groups of isolates could be observed by cluster analysis based on Sheath's dissimilarity criterion at 66% and using the Average Fungal Respiration Intensity (AFRI; Fig. 7A) and the Average Fungal Growth Intensity (AFGI; Fig. 7B). As indicated by previous analyses, the response to these treatments by the G150/14 isolate differed from that of the other tested isolates (as evident in Fig. 7A,B). This underscores the significance of intraspecific differences when evaluating the effects of specific treatments on organisms. Furthermore, consistent separation of fungal biomass utilization and growth under exposure to the natural extracts was observed for all other isolates. Two isolates (G160/14 and G135/14), were characterized by a uniform response and confirming their inclusion in the previously mentioned high resistance group (referred to as the V group). The remaining isolates (G154/14, G129/14, G132/14, G151/14, G130/14, G226/14 and G127/14) were distributed into a third cluster based both on AFRI and AFGI.

From this research, three groups of isolates with different sensitivity to plant extracts could be distinguished, including those characterized by high (G150/14), medium (G126/14, G127/14, G129/14, G130/14, G132/14, G151/14, G154/14) and low (G135/14, G160/14) sensitivity to the tested dry extracts and plant oils.

### Metabolic profile analysis—changes after pre-incubation with marigold extract

**Figure 8:**
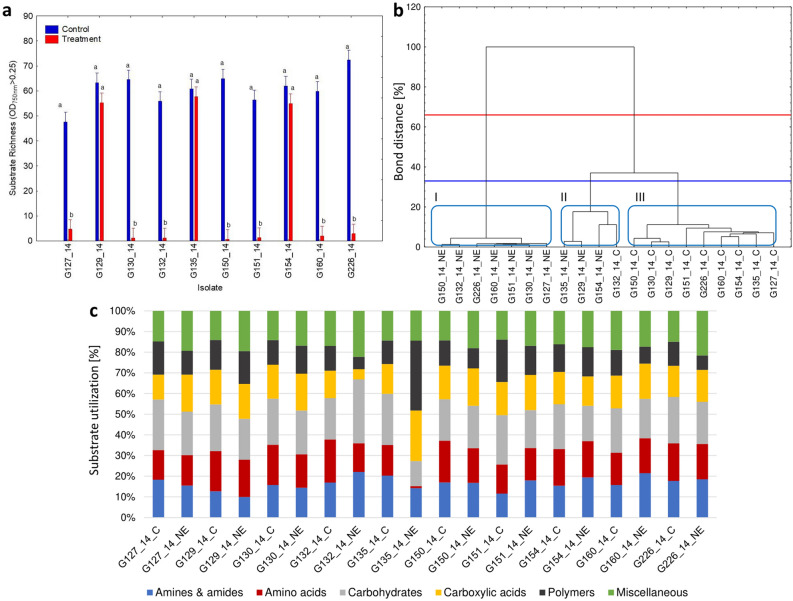
The effect of marigold extract on *Neosartorya* spp. metabolic profiles. Substrate richness per isolate (**a**). Cluster analysis of fungal biomass production similarity between different isolates of *Neosartorya* spp. pre-cultured in the presence of marigold extract dilution (NE) and without it (C) after 120 h of incubation, with the lines representing the Sneath criterion at 33% (blue line) and at 66% (red line) and numbers I, II, III for observable clusters (**b**). Utilization of carbon source groups by isolates treated (NE) and untreated (C) with marigold extract after 120 h of incubation (c).

Pre-incubation of isolates with marigold extract (150 µg mL^−1^) led to different behaviours amongst the analysed isolates. Over half of the isolates (seven out of ten) exhibited a significant loss of substrate richness of metabolized substances compared to the control (Fig. 8A), while the remaining three isolates (G129/14, G135/14, and G154/14) exhibited a significant loss of substrate richness (R index) of metabolized substances compared to the control. Cluster analysis (Fig. 8B) showed a clear division between isolates that had been pre-incubated on marigold extract and control isolates that were cultured on PDA medium in their biomass production abilities. Only 3 isolates (G129/14, G135/14, and G154/14) exposed to marigold extract were found to cluster with isolates not exposed to this plant extract. The distinct response of these three isolates after pre-culturing in marigold extract is also depicted in Fig. 8A. In general, the isolates clustered as 3 separate groups according to the Sneath criterion (33%), with one group representing the control, the second made up of the treatment group and a third containing a few isolates from both the treatment and control groups. The utilization percentages within the six groups of analysed substances using the FF microplate showed variations in utilization patterns based on isolates’ pre-incubation conditions (Fig. 8C). A notable increase in average utilization of the group of miscellaneous substrates (referred to miscellaneous group from now on) was observed for nearly all isolates that had been pre-incubated with marigold extract. In the case of G135/14 (characterised by the lowest sensitivity to plant extracts), significant changes were observed when compared to the control. These changes included the loss of substrate utilization within the amino acids group, a reduction in utilization within the carbohydrates group, and, nearly double the utilization within the carboxylic acids and polymers groups. In the case of the G150/14 isolate, characterized by the highest sensitivity to the tested plant extracts, pre-incubation with marigold extract caused an increase uptake of miscellaneous group substrates, but it did not cause a change in the use of individual groups of carbon substrates compared to the control fungi, with only a very slight decrease of amino acids and polymer metabolism visible. In the remaining isolates, characterized by medium sensitivity to the tested plant extracts, when treated with marigold extract there was generally a lower use of amines, amides and carbohydrates, as well as an increased use of compounds belonging to the miscellaneous group. G150/14 may be more sensitive than G135/14 and the rest of the more resilient isolates (e.g. G129/14) because it maintains a more balanced utilization of carbon substrates even when exposed to marigold extract, whereas G135/14 exhibits significant changes in substrate utilization when compared to the control. Thus isolate G150/14 possibly exhibits an inability to adapt and change its metabolism in stressful conditions. The obtained results indicate that the metabolic properties of fungi of the genus *Neosartorya* have an impact on shaping their resistance to plant extracts. Especially the high use of nitrogen compounds, in particular the proportionally equal ratio of the use of amines, amides and amino acids, suggests a high sensitivity of fungal isolates to plant extracts. However, the predominance of the use of carboxylic acids by fungal isolates may suggest their lower sensitivity to the tested plant extracts.

**Figure 9:**
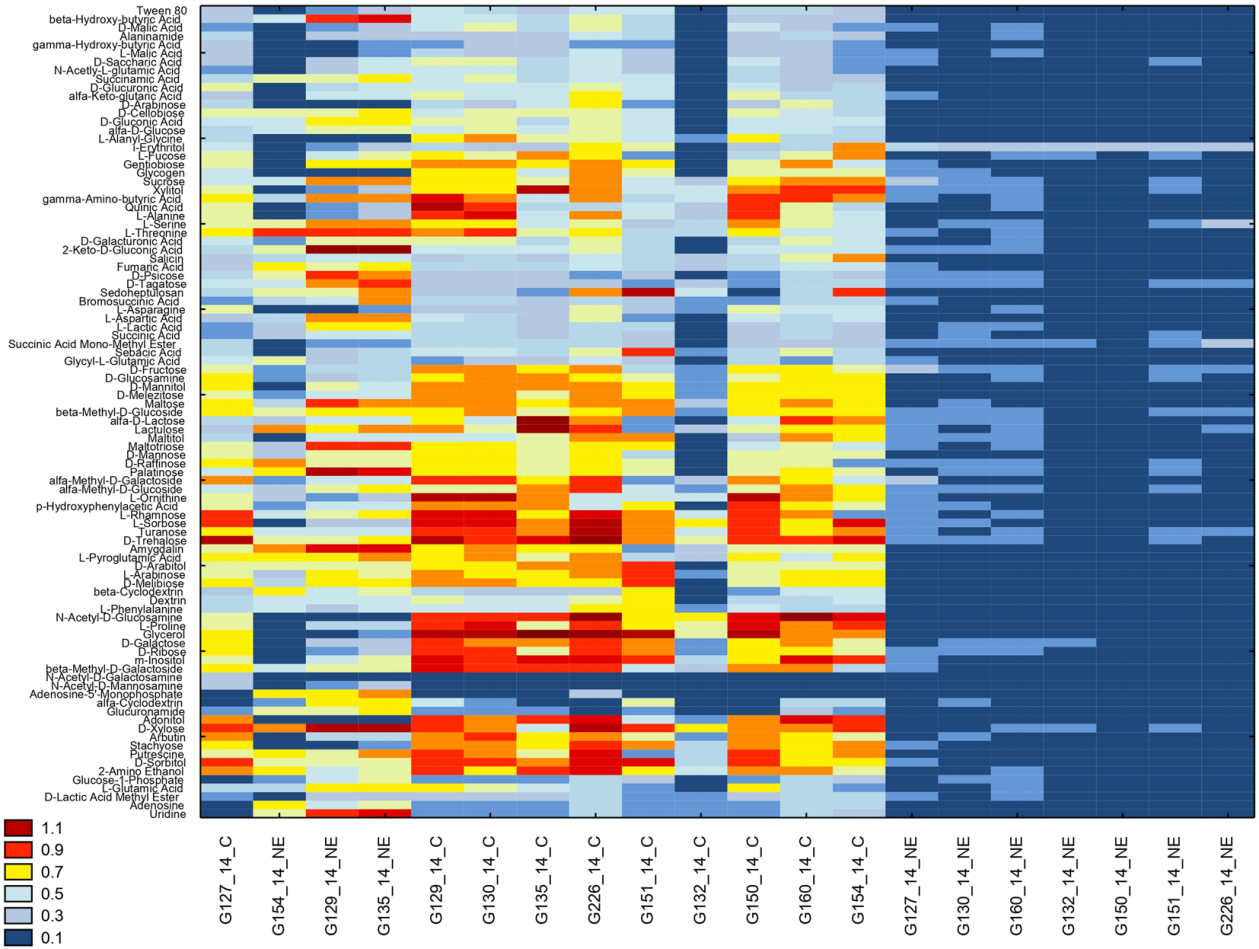
*Neosartorya* spp. biomass production on the FF microplate. Isolates cultured in the presence of marigold extract (NE) and without it (C) after 120 h of incubation.

Disparities among isolates were also evident in the heatmap analysis of the values related to fungal biomass production (corresponding to the optical density at 750 nm) as shown in Fig. 9. *Neosartorya* spp. grown without the marigold extract (control group) showed the highest growth in the presence of l-rhamnose, d-xylose, d-sorbitol, *N*-acetyl-d-glucosamine, adonitol, maltose and d-trehalose. Despite it not inducing the highest growth in the control group, i-erythritol emerged as the only carbon source universally promoting growth of *Neosartorya* spp. incubated on media with marigold extract. d-Mannitol was responsible for a moderate production of fungal biomass (ranging from 0.7 to 0.9) in isolates obtained from strawberry fruits, such as G129/14, G130/14, G135/14, and G226/14. Meanwhile, d-trehalose yielded positive results across almost all fungal isolates pre-incubated without the addition of marigold extract, resulting in optical density values between 0.7 and 1.1. Three isolates possessed a somewhat similar growth capacity to the control (G154/14, G129/14, G135/14), although it was slightly diminished. Such similarity could be a sign of high resistance of three isolates to incubation with marigold extract. The three isolates differed from the control group most notably in their inability to metabolise *N*-acetyl-d-glucosamine and adonitol which were both well metabolised by the isolates cultivated on PDA media without marigold extract.

### Scanning electron and fluorometric microscopy analyses

**Figure 10:**
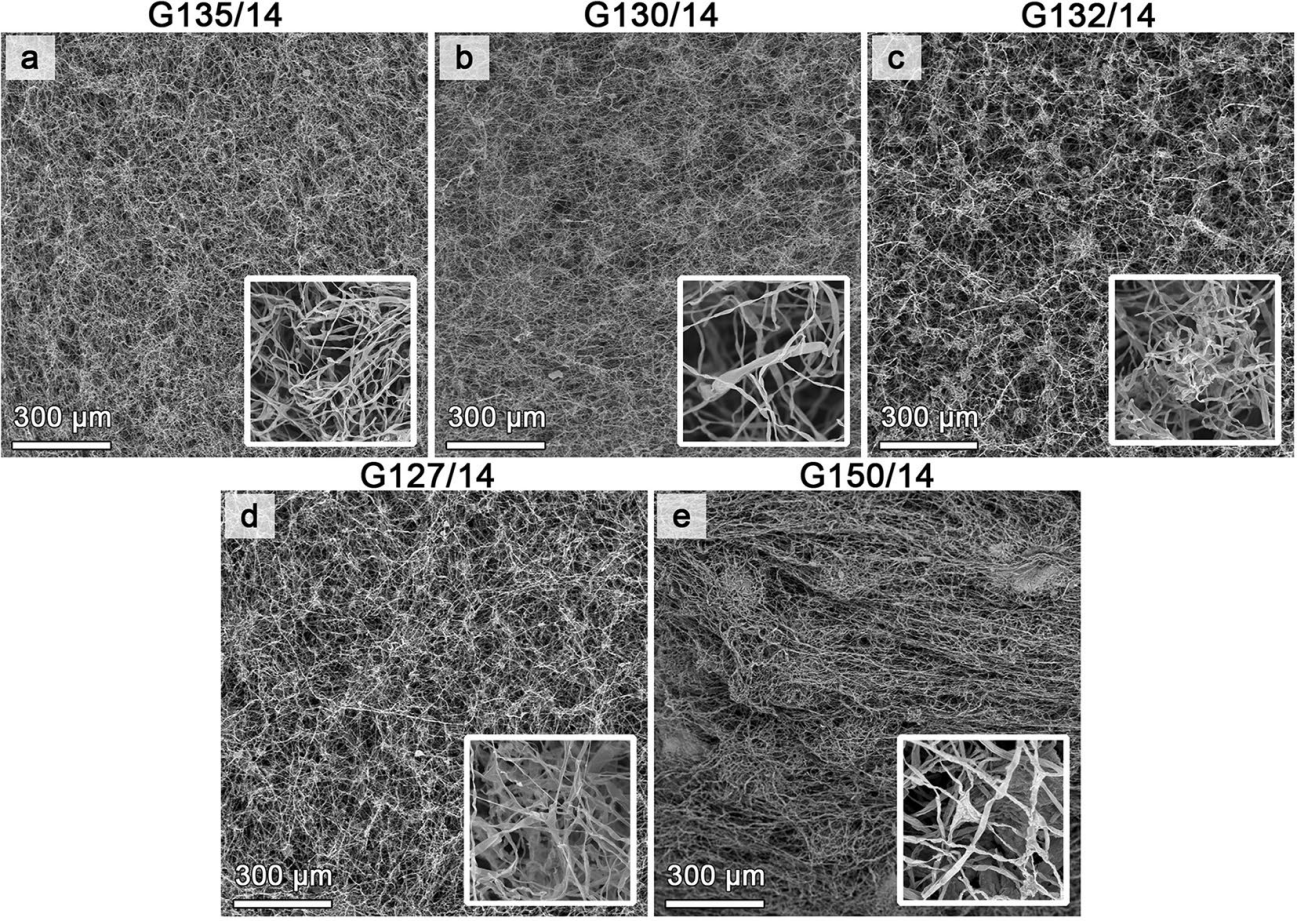
*Neosartorya* spp. observation using SEM – isolates G135/14 (**a**), G130/14 (**b**), G132/14 (**c**), G127/14 (**d**), G150/14 (**e**) cultivated on PDA media, 200X magnification and 5000X magnification zoom.

When observed under a scanning electron microscope (SEM), mycelium of *Neosartorya* isolates appeared as a complex network of branching, thread-like structures. The mycelium displayed a high interconnectivity, with numerous fine hyphae extending in multiple directions (Fig. 10). Hyphae were generally characterized by a smooth surface and varied from 1.5 to 5 µm in width. The mycelium of isolates G135/14 (the least sensitive) and G130/14 (an isolate at the lower end of average sensitivity) appeared more dense and tightly woven, (Fig. 10A,B) than the mycelium of isolates G132/14 (average sensitivity) and G127/14 (high sensitivity) (Fig. 10C–E). Additionally, after the 10 day long incubation period, isolates produced well-developed ascospores (Fig. S5).

**Figure 11:**
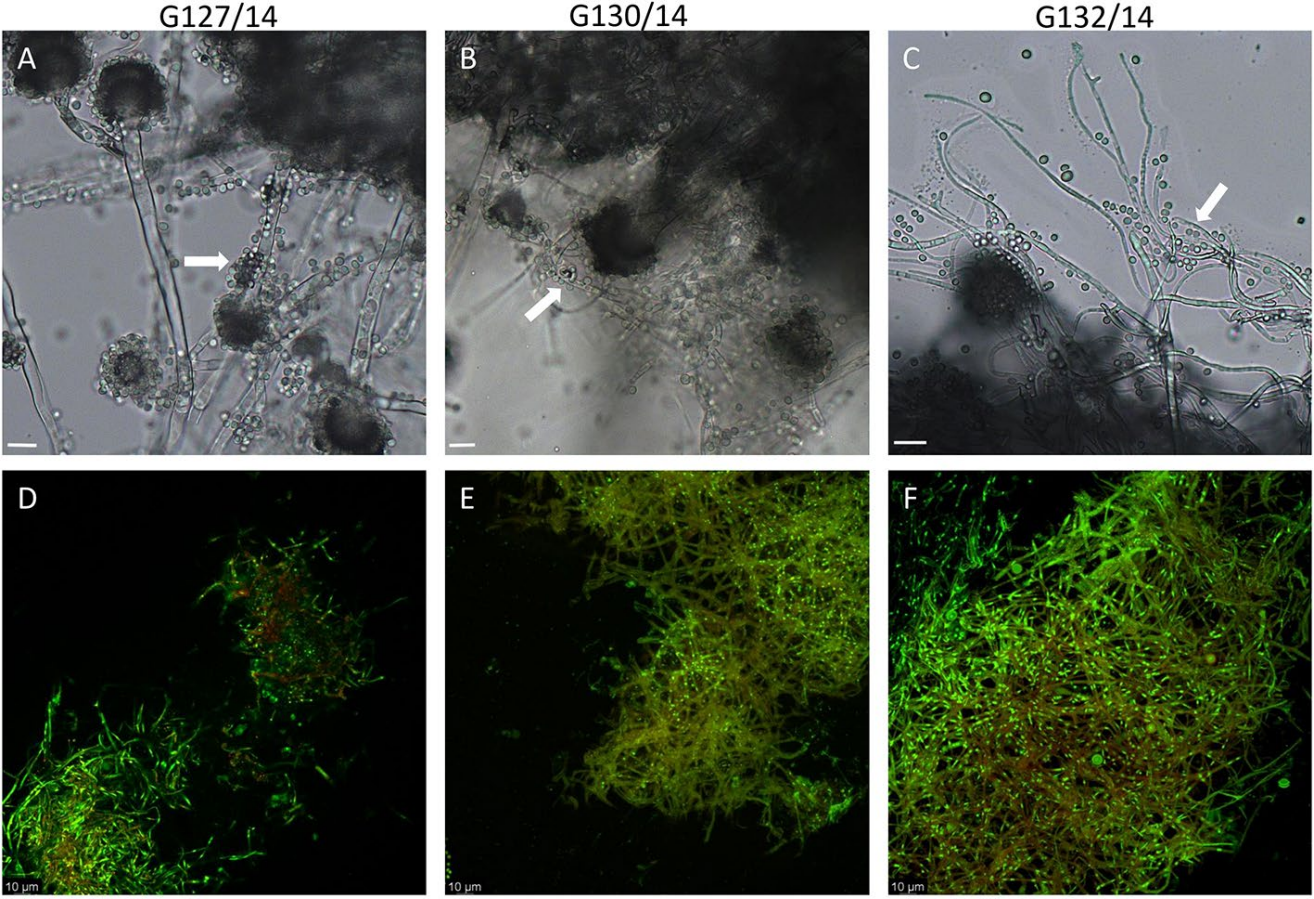
Observation of *Neosartorya* spp. isolates. G127/14 (**A**, **D**), G130/14 (**B**, **E**) and G132/14 (**C**, **F**) through optical microscope (**A**, **B**, **C**; 40X magnification) and fluorescence imaging (**D**, **E**, **F**). Visible spores are indicated by white arrows.

Optical imaging after 5 days of incubation showed that isolate G127/14 had the most prominent conidia and conidiospore formation (Fig. 11A), G130/14 possessed a moderate amount of conidia (Fig. 11B) and G132/14 had the least visible conidia from all 3 isolates and exhibited a slower rate of growth compared to the other isolates (Fig. 11C). During fluorescence imaging withFUN-1 dye, no significant differences in filament viability were observed among the isolates (Fig. 11D–F).

## Discussion

*Neosartorya* spp. is a pathogenic fungus capable of affecting fruit prior to harvesting, potentially leading to contamination of finished products (such as juice and preserved fruit) through spores and mycotoxins^7^. So far, studies have focused on traditional food protection techniques used during manufacture, such as modifying sugar content, temperature or pressure of processing^51,52^. However the use of such methods poses a risk of altering the physical properties of food, leading to a decrease in value and consumer dissatisfaction^17^. Therefore it is essential to search for alternative, health-positive or neutral methods of protection that are easy to apply in high-scale farming. Examples of effective fungicides that could be directly applied to the general market are antagonistic organisms or natural extracts, both easily applied e.g. in form of sprays^53,54^. In our research, aside from natural dry powder solutions, water emulsions of oils were used as antifungal agents.

Ben Miri et al.^55^ reported sensitivity of *Aspergillus ochraceus* and *A*. *niger* to menthol and eugenol, common constituents derived from mint or myrtle. Some reports also focus on reduction in aflatoxin synthesis, spore germination and mycelium growth of *Aspergillus flavus* under the influence of natural extracts (e.g. Shirazi thyme and cumin seed)^55^. In our research, natural extracts (lavender and marigold dry extracts; lavender, tea tree, clove and rosemary oils) caused an inhibition of *Neosartorya* sp. isolates’ mycelium growth. Overall, all these substances exhibited an inhibitory effect on the growth of fungal mycelium at low concentrations, and as the concentrations increased, a nearly exponential rise in this effect was noted (Fig. 5A). At all concentrations, the size of the inhibition zones were relatively consistent. However, when the concentration of 1000 μg mL^−1^ was used, a significant and distinct separation into three groups was evident. Clove oil was particularly effective, followed by lavender and tea tree oil, with a lesser effect observed with lavender flower and marigold extracts, and rosemary oil. While their impact on mycotoxin production and thermal resistance remains unknown, our experiments confirmed the antifungal ability of these extracts, expressed as growth inhibition. In general, essential oils are considered more potent than dry extracts, albeit less stable due to evaporation^56^, potentially due to the use of different extraction methods to generate these substances. Natural extracts are obtained using common solvents, but the choice of solvent and extraction conditions can be critical factors that could affect the yield and composition of the active ingredients extracted from different plant parts^22^. A more detailed examination of Fig. 4A revealed that the division of fungal isolates into five groups based on their sensitivity after incubation with the analysed natural substances closely aligned to their origin. Isolates G150/14, G154/14, G159/14, and G151/14, which were obtained from soil, displayed greater sensitivity to incubation with these natural substances. In contrast, the more resistant strains, G131/14, G130/14, G132/14, G253/14, G239/14, G135/14, and G129/14, were all isolated from strawberry fruit. This differential response could be attributed to the limited resources available in soil, hindering their growth and development across all growth stages. Soil, however, could also have served as a protective environment, shielding the fungus from external changes and events. Conversely, strawberry fruit harbours a wider array of carbon resources for growth, but the sudden change in these resources could have compelled these fungi to exhibit metabolic plasticity. This would have resulted in immediate metabolic adjustments to cope with environmental fluctuations, ensuring the survival of the fungal mycelium.

The inhibitory effect of the analysed natural substances on *Neosartorya* sp. growth was further confirmed by the MT2 microplate analysis. The use of MT2 microplates determined whether various *Neosartorya* isolates utilized these natural resources for their metabolism and if they subsequently exhibited resistance or sensitivity to these substances. All natural extracts caused a substantial decrease in biomass production (Fig. 6A). A strong inhibitory effect was observed in response to the lavender extract, rosemary, and lavender oils. Interestingly, the isolates underwent a significant decrease in mycelium production at concentrations of 5 and 150 µg mL^−1^ for all oils and dry extracts, instead of being strongly affected by the highest concentration. It's important to note that the dose–response relationship and optimal concentrations can vary depending on the specific natural extract. It is plausible that the 5 µg mL^−1^ concentration resulted in a higher solute “liquidity” within the solvent (distilled and sterilized water in our case), facilitating the passage of natural substances into the fungal mycelium, thereby eliciting negative reactions in fungal metabolism, ultimately preventing mycelium proliferation by the analysed substance. In contrast, at concentrations of 1000 µg mL^−1^, the solute may be less “fluid,” reducing penetration of the substances into the fungus, resulting in a less pronounced effect compared to the minimum concentration analysed. *Neosartorya* spp. may also have a susceptibility threshold, beyond which higher doses have limited additional inhibitory effects. Thus the toxicity-growth inhibition relationship may be non-linear, with higher concentrations exhibiting increased toxicity but diminished inhibitory effects.

We observed that at concentrations of 5 and 150 µg mL^−1^ (causing the most significant antifungal effect on growth) the AFRI/AFGI ratio was lower for the majority of treatments than for the control (0 µg mL^−1^). However, these values were higher than the control for rosemary oil (at both concentrations) and for lavender and marigold extract at 150 µg mL^−1^ (Fig. 6B). Considering that the AFRI/AFGI ratio can provide insights into whether the isolates are in a state of stress^47^, we can conclude that the fungus exhibits the most evident stress response when incubated with marigold extract (ranging from 50 to 1000 μg mL^−1^) and lavender oil at 1000 μg mL^−1^. This suggests that exposure of *Neosartorya* sp. to marigold extract negatively affects fungal biomass production (Fig. 6A) and induces a state of stress, manifested through an inefficient metabolism (Fig. 6B). There was, however, high variability between individual *Neosartorya* spp. isolates (Fig. 8) to the treatments. Focusing specifically on the AFGI, only one isolate (G130/14) displayed a lower growth intensity in the control compared to the natural treatments analysed, and isolate G132/14 showed no difference in growth between the control and the treatment. The remaining eight isolates exhibited a negative effect on fungal biomass production during the incubation period with the different natural substances analysed (Fig. 8A), contributing to a lower stress condition (Fig. 8B). Analysis using MT2 microplates highlighted the changes in the metabolic level of *Neosartorya* sp. due to incubation with these natural substances, rendering fungi unable to use the substances effectively, resulting in significantly lower growth compared to the control incubated under favourable conditions. Furthermore, the substantial variation in behaviour among the various isolates (Fig. 8) corresponds with the findings of Panek et al.^57^. These authors investigated the chemical sensitivity, using Biolog™ Phenotype MicroArray analysis, of two different isolates of *Neosartorya fischeri* from distinct environments: one isolated from canned apples in 1923 and another from thermally processed strawberry products in 2012. Their study revealed that the fungus isolated from the processed strawberry product demonstrated much greater resistance to the chemicals analysed through the PM platform compared to the isolate from canned apples. Despite the similar isolation and storage time of isolates studied in our experiments, many of the fungi derived from strawberry fruit, possessed an increased resistance to stress conditions compared to those from soil. Three isolates (G130/14 and G226/14, both resistant, and G132/14, displaying no effect in this study) originating from strawberry fruit exhibited greater resistance when incubated with natural substances. This supports the results obtained from the inhibition test and confirms that *Neosartorya* spp. isolates from strawberry fruits are more resistant to incubation with these natural substances. When analysing how the metabolism of *Neosartorya* spp. isolates could be influenced (FF microplates) by pre-cultivation in marigold extract (Fig. 9), a clear division into two clusters (Fig. 9B) was noticed, based on whether the isolates were pre-incubated with or without marigold extract. Within this separation, one exception was noted for three isolates pre-incubated in marigold (G135/14, G129/14, and G154/14), which were grouped with isolates pre-incubated without the natural extract. For these three isolates, incubation with marigold extract did not result in negative effects on metabolism, in contrast to the majority of the analysed isolates. Instead, it led to a significant increase in substrate richness (Fig. 8A) compared to the control. Furthermore, as observed in the other analyses we conducted, the isolates that were not affected by incubation with these natural substances were found to be fungi isolated from strawberry fruits, specifically G135/14 and G129/14. Upon examining the optical density values obtained at 750 nm (Fig. 10), it was apparent that the solutes involved in acquiring heat resistance capacity and the maturation of ascospores^14,16,58^ allowed for a high production of fungal mycelium in only a few fungal isolates that were pre-incubated without marigold extract. These findings offer potential regarding the application of marigold extract as a natural fungicide. Five days of pre-incubation with this natural substance led to a distinct alteration of metabolism, limiting the production of fungal biomass across 95 different carbon substrates. Additionally, only three isolates pre-incubated with marigold exhibited increased fungal biomass compared to the control for specific carbon resources, including β-hydroxy-butyric acid, l-serine, 2-keto-d-gluconic acid, fumaric acid, d-tagatose, l-aspartic acid, palatinose, and uridine.

Upon analysing the mycelium structure of five *Neosartorya* isolates grown in a standard culture medium (PDA), it was clear that isolate G150/14 (originating from soil) exhibited a distinctive structure compared to the other isolates (Fig. 11). This structural variation in the mycelium could potentially account for the differential response to the treatments examined in this study.

Considering the cumulative results across the various treatments and dosages, it seems clear that both the dry extracts and natural oils exert an inhibitory influence on the growth and development of *Neosartorya* spp. fungal mycelia. Furthermore, pre-incubation with marigold extract results in a substantial reduction in the growth of fungal mycelium across different carbon substrates, effectively slowing its growth on d-mannitol and d-trehalose, both of which are highly soluble compounds associated with the acquisition of heat resistance by *Neosartorya* spp.^59,60^. These findings are a cause for optimism regarding the prospective application of these natural substances to mitigate the presence and proliferation of fungi in fruit-derived products. Moreover, this study highlights that fungi isolated from strawberry fruits demonstrate greater resistance compared to those isolated from soil, which warrants further investigation in the future. This discrepancy is further supported by the visualization of fungal mycelium structure using a scanning electron microscope (SEM). The variance in treatment response is likely linked to potential intraspecific differences within the *Neosartorya* genus, prompting an additional need for further exploration.

## Conclusions

This is a first report on the effect of natural extracts on *Neosartorya* spp. utilizing both inhibition tests and microplate metabolic assays. Using these methodologies we were able to identify variation in the susceptibility of individual isolates to the effects of plant extracts and establish 3 main groups of increasing sensitivity, underlining the possibility of a non-uniform response to stress factors amongst isolates of the same genus. There seems to be a link between innate fungal substrate utilization abilities (which vary between isolates) and sensitivity to natural extracts, with weaker isolates naturally preferring nitrogen compounds and more resilient ones preferring carboxylic acids. Another observable difference between isolates was the mycelial density, where more resistant isolates were characterized by denser mycelium than sensitive fungi. The results indicate that natural extracts, especially lavender oil, yield an effective outcome in growth inhibition of fungi belonging to *Neosartorya* genus. To conclude, an in-depth examination of *Neosartorya* spp. was conducted, focusing on metabolic capabilities under the influence of a natural extracts possessing antimicrobial capabilities. Information on fungal phenotypes and specific nutrient utilization profiles was also provided. These findings are very important for understanding functional and morphological features in shaping the resistance of *Neosartorya* spp. to plant extracts. Moreover, they could be used to develop better prevention and intervention methods against *Neosartorya* spp., and in the development of new active compounds for the control of microbiological food contaminants and pathogenic species, which is important for the development of a more sustainable agriculture. However, further research is required to determine the underlying cause of such metabolic diversity among the tested *Neosartorya* spp. isolates.

### Supplementary Information

Supplementary Information.

## Data Availability

The datasets used and/or analysed during the current study are available from the corresponding author on reasonable request.

## References

[1] El Khetabi A, Lahlali R, Ezrari S, Radouane N, Lyousfi N, Banani H (2022). Role of plant extracts and essential oils in fighting against postharvest fruit pathogens and extending fruit shelf life: A review. Trends Food Sci. Technol..

[2] Snyder AB, Worobo RW (2018). Fungal spoilage in food processing. J. Food Prot..

[3] Tournas V, Traxler RW (1994). Heat resistance of a *Neosartorya fischeri* strain isolated from pineapple juice frozen concentrate. J. Food Prot..

[4] Yaguchi T, Imanishi Y, Matsuzawa T, Hosoya K, Hitomi J, Nakayama M (2012). Method for identifying heat-resistant fungi of the genus *Neosartorya*. J. Food Prot..

[5] Brienzo M, Arantes V, Milagres AM (2008). Enzymology of the thermophilic ascomycetous fungus *Thermoascus aurantiacus*. Fungal Biol. Rev..

[6] Houbraken J, Giraud S, Meijer M, Bertout S, Frisvad JC, Meis JF, Bouchara JP, Samson RA (2013). Taxonomy and antifungal susceptibility of clinically important *Rasamsonia* species. J. Clin. Microbiol..

[7] Chen S, Fan L, Song J, Zhang H, Doucette C, Hughes T, Campbell L (2022). Quantitative proteomic analysis of *Neosartorya pseudofischeri* ascospores subjected to heat treatment. J. Proteom..

[8] Górska EB, Stępien W, Cunha A, Sierra-Garcia IN, Szyszkowska K, Gozdowski D (2022). Microbial diversity as an indicator of a diversified cropping system for luvisols in a moderate climate. Case study—Long term experiments from Poland. Ecol. Indic..

[9] de Sá JDM, Kumla D, Dethoup T, Kijjoa A (2022). Bioactive compounds from terrestrial and marine-derived fungi of the genus *Neosartorya*. Molecules.

[10] Bang S, Song JH, Lee D, Lee C, Kim S, Kang KS, Lee JH, Shim SH (2019). Neuroprotective secondary metabolite produced by an endophytic fungus, *Neosartorya fischeri* JS0553, isolated from *Glehnia littoralis*. J. Agric. Food Chem..

[11] Igbinigie EE, Atkins S, van Breugel Y, van Dyke S, Davies-Coleman MT, Rose PD (2008). Fungal biodegradation of hard coal by a newly reported isolate, *Neosartorya*
*fischeri*. Biotechnol. J..

[12] Shin HD, McClendon S, Le T, Taylor F, Chen RR (2006). A complete enzymatic recovery of ferulic acid from corn residues with extracellular enzymes from *Neosartorya spinosa* NRRL185. Biotechnol. Bioeng..

[13] Tóth L, Poór P, Ördög A, Váradi G, Farkas A, Papp C, Bende G, Tóth GK, Rákhely G, Marx F, Galgóczy L (2022). The combination of *Neosartorya (Aspergillus) fischeri* antifungal proteins with rationally designed γ-core peptide derivatives is effective for plant and crop protection. Biocontrol.

[14] Hocking AD (2006). 17—*Aspergillus* and related teleomorphs. Food Spoilage Microorganisms.

[15] de Cássia Martins Salomão B, Rajauria G, Tiwari BK (2018). Chapter 16—Pathogens and spoilage microorganisms in fruit juice: An overview. Fruit Juices.

[16] Wyatt, T. T. Mechanisms underlying extreme heat resistance of ascospores of *Neosartorya fischeri*. Doctoral dissertation, Uitgeverij Merlin (2014).

[17] Splittstoesser DF, Nielsen P, Churey JJ (1993). Detection of viable ascospores of *Neosartorya*. J. Food Prot..

[18] Frac M, Jezierska-Tys S, Yaguchi T (2015). Occurrence, detection, and molecular and metabolic characterization of heat-resistant fungi in soils and plants and their risk to human health. Adv. Agron..

[19] Fornal E, Parfieniuk E, Czeczko R, Bilinska-Wielgus N, Frac M (2017). Fast and easy liquid chromatography–mass spectrometry method for evaluation of postharvest fruit safety by determination of mycotoxins: Fumitremorgin C and verruculogen. Postharvest Biol. Technol..

[20] Nielsen PV, Beuchat LR, Frisvad JC (1989). Growth and fumitremorgin production by *Neosartorya fischeri* as affected by food preservatives and organic acids. J. Appl. Bacteriol..

[21] European Commission. Farm to Fork Strategy for a fair, healthy and environmentally-friendly food system. https://ec.europa.eu/food/sites/food/files/safety/docs/f2f_action-plan_2020_strategy-info_en.pdf (2020).

[22] Matrose NA, Obikeze K, Belay ZA, Caleb OJ (2021). Plant extracts and other natural compounds as alternatives for post-harvest management of fruit fungal pathogens: A review. Food Biosci..

[23] Mutlu-Ingok A, Devecioglu D, Dikmetas DN, Karbancioglu-Guler F, Capanoglu E (2020). Antibacterial, antifungal, antimycotoxigenic, and antioxidant activities of essential oils: An updated review. Molecules.

[24] Abdolahi A, Hassani A, Ghosta Y, Javadi T, Meshkatalsadat MH (2010). Essential oils as control agents of postharvest *Alternaria* and *Penicillium* rots on tomato fruits. J. Food Saf..

[25] Olea AF, Bravo A, Martínez R, Thomas M, Sedan C, Espinoza L, Zambrano E, Carvajal D, Silva-Moreno E, Carrasco H (2019). Antifungal activity of eugenol derivatives against *Botrytis cinerea*. Molecules.

[26] Wang C, Zhang J, Chen H, Fan Y, Shi Z (2010). Antifungal activity of eugenol against *Botrytis cinerea*. Trop. Plant Pathol..

[27] Zhang H, Kalhoro MT, Huo D, Faqir Y, Nabi F, Wang F, Gao Z, Chen T (2023). Screening antifungal properties of essential oils against taro leaf blight disease. J. Plant Dis. Prot..

[28] Amiri A, Dugas R, Pichot AL, Bompeix G (2008). In vitro and in vivo activity of eugenol oil (*Eugenia caryophylata*) against four important postharvest apple pathogens. Int. J. Food Microbiol..

[29] Tripathi P, Dubey NK, Shukla AK (2008). Use of some essential oils as post-harvest botanical fungicides in the management of grey mould of grapes caused by *Botrytis cinerea*. World J. Microbiol. Biotechnol..

[30] Zabka M, Pavela R, Gabrielova-Slezakova L (2011). Promising antifungal effect of some Euro-Asiatic plants against dangerous pathogenic and toxinogenic fungi. J. Sci. Food Agric..

[31] Pesis E (2005). The role of the anaerobic metabolites, acetaldehyde and ethanol, in fruit ripening, enhancement of fruit quality and fruit deterioration. Postharvest Biol. Technol..

[32] Jin X, Wu Y, Santhamoorthy M, Le TTN, Yuan Y, Xia C (2022). Volatile organic compounds in water matrices: Recent progress, challenges, and perspective. Chemosphere.

[33] Ho PL, Tran DT, Hertog ML, Nicolaï BM (2021). Effect of controlled atmosphere storage on the quality attributes and volatile organic compounds profile of dragon fruit (*Hylocereus undatus*). Postharvest Biol. Technol..

[34] Saltveit ME, Mencarelli F (1988). Inhibition of ethylene synthesis and action in ripening tomato fruit by ethanol vapors. J. Am. Soc. Hortic. Sci..

[35] Raymundo LC, Griffiths AE, Simpson KL (1970). Biosynthesis of carotenoids in the tomato fruit. Phytochemistry.

[36] Pertile G, Panek J, Oszust K, Siczek A, Frac M (2018). Intraspecific functional and genetic diversity of *Petriella setifera*. PeerJ.

[37] Camacho C, Coulouris G, Avagyan V, Ma N, Papadopoulos J, Bealer K, Madden TL (2009). BLAST+: Architecture and applications. BMC Bioinform..

[38] Malarczyk DG, Panek J, Frąc M (2020). Triplex real-time PCR approach for the detection of crucial fungal berry pathogens—*Botrytis* spp., *Colletotrichum* spp., and *Verticillium* spp. Int. J. Mol. Sci..

[39] White, T. J., Bruns, T., Lee, S. & Taylor, J. Amplification and direct sequencing of fungal ribosomal RNA genes for phylogenetics. In *PCR Protocols* 315–322. 10.1016/b978-0-12-372180-8.50042-1 (1989).

[40] Tamura K, Stecher G, Kumar S (2021). MEGA11: Molecular evolutionary genetics analysis version 11. Mol. Biol. Evol..

[41] Pertile G, Frąc M (2023). The antifungal effect of pyroligneous acid on the phytopathogenic fungus Botrytis cinerea Int. J. Mol. Sci..

[42] Frac M, Gryta A, Oszust K, Kotowicz N (2016). Fast and accurate microplate method (Biolog MT2) for detection of *Fusarium* fungicides resistance/sensitivity. Front. Microbiol..

[43] Oszust K, Panek J, Pertile G, Siczek A, Oleszek M, Frac M (2018). Metabolic and genetic properties of *Petriella setifera* precultured on waste. Front. Microbiol..

[44] Preston-Mafham J, Boddy L, Randerson PF (2002). Analysis of microbial community functional diversity using sole-carbon-source utilization profiles—A critique. FEMS Microbiol. Ecol..

[45] Dobranic JK, Zak JC (1999). A microtiter plate procedure for evaluating fungal functional diversity. Mycologia.

[46] Frąc M (2012). Mycological evaluation of dairy sewage sludge and its influence on the functional diversity of soil microorganisms. Acta Agrophys..

[47] Pinzari F, Ceci A, Abu-Samra N, Canfora L, Maggi O, Persiani A (2016). Phenotype MicroArray™ system in the study of fungal functional diversity and catabolic versatility. Res. Microbiol..

[48] Mącik M, Gryta A, Sas-Paszt L, Frąc M (2023). New insight into the soil bacterial and fungal microbiome after phosphorus biofertilizer application as an important driver of regenerative agriculture including biodiversity loss reversal and soil health restoration. Appl. Soil Ecol..

[49] Frąc M, Oszust K, Lipiec J (2012). Community level physiological profiles (CLPP), characterization and microbial activity of soil amended with dairy sewage sludge. Sensors.

[50] Pawlik A, Ruminowicz-Stefaniuk M, Frąc M, Mazur A, Wielbo J, Janusz G (2019). The wood decay fungus *Cerrena unicolor* adjusts its metabolism to grow on various types of wood and light conditions. PLoS One.

[51] Berni E, Tranquillini R, Scaramuzza N, Brutti A, Bernini V (2017). *Aspergilli* with *Neosartorya*-type ascospores: Heat resistance and effect of sugar concentration on growth and spoilage incidence in berry products. Int. J. Food Microbiol..

[52] Evelyn Kim HJ, Silva FVM (2016). Modeling the inactivation of *Neosartorya fischeri* ascospores in apple juice by high pressure, power ultrasound, and thermal processing. Food Control.

[53] Podgórska-Kryszczuk I (2023). Biological control of *Aspergillus flavus* by the yeast *Aureobasidium pullulans* in vitro and on tomato fruit. Plants.

[54] Podgórska-Kryszczuk I, Pankiewicz U (2023). Assessment of the fungistatic properties of *Calendula*
*officinalis* L. water extract and the effect of its addition on the quality of wheat bread. Appl. Sci..

[55] Ben Miri Y, Nouasri A, Herrera M, Djenane D, Ariño A (2023). Antifungal activity of menthol, eugenol, and their combination against *Aspergillus ochraceus* and *Aspergillus niger* in vitro and in stored cereals. Foods.

[56] Tavakolipour H, Kalbasi-Ashtari A, Mokhtarian M (2020). Effects of coating pistachio kernels with mixtures of whey protein and selected herbal plant extracts on the growth inhibition of *Aspergillus flavus* and prevention of aflatoxin during storage. J. Food Saf..

[57] Oikeh EI, Oviasogie FE, Omoregie ES (2020). Quantitative phytochemical analysis and antimicrobial activities of fresh and dry ethanol extracts of *Citrus*
*sinensis* (L.) Osbeck (sweet Orange) peels. Clin. Phytosci..

[58] Panek J, Frąc M, Bilińska-Wielgus N (2016). Comparison of chemical sensitivity of fresh and long-stored heat-resistant *Neosartorya fischeri* environmental isolates using Biolog Phenotype MicroArray system. PLoS One.

[59] Wyatt TT, van Leeuwen MR, Wösten HA, Dijksterhuis J (2014). Mannitol is essential for the development of stress-resistant ascospores in *Neosartorya fischeri* (*Aspergillus fischeri*). Fungal Genet. Biol..

[60] Wyatt TT, van Leeuwen MR, Golovina EA, Hoekstra FA, Kuenstner EJ, Palumbo EA, Dijksterhuis J (2015). Functionality and prevalence of trehalose-based oligosaccharides as novel compatible solutes in ascospores of *Neosartorya fischeri* (*Aspergillus fischeri*) and other fungi. Environ. Microbiol..

