# Supplementary material for: Comprehensive antifungal investigation of natural plant extracts against Neosartorya spp. (Aspergillus spp.) of agriculturally significant microbiological contaminants and shaping their metabolic profile
Source: Sci Rep. 2024 Apr 10;14:8399. doi: 10.1038/s41598-024-58791-4 (PMC11006677; doi:10.1038/s41598-024-58791-4)
Supplement: Supplementary file 1 — Supplementary Information. [file 41598_2024_58791_MOESM1_ESM.docx]

**Comprehensive antifungal investigation of natural plant extracts against *Neosartorya* spp. (*Aspergillus* spp.) of agriculturally significant microbiologial contaminants and shaping their metabolic profile**

Wiktoria Maj^1^, Giorgia Pertile^1^, Sylwia Różalska^2^, Kamil Skic^1^, Magdalena Frąc^1*^

**^1^** Institute of Agrophysics, Polish Academy of Sciences, Doświadczalna 4, 20-290 Lublin, Poland

**^2^** University of Łódź, Narutowicza 68, 90-136 Łódź, Poland

*Corresponding author: [m.frac@ipan.lublin.pl](mailto:m.frac@ipan.lublin.pl)


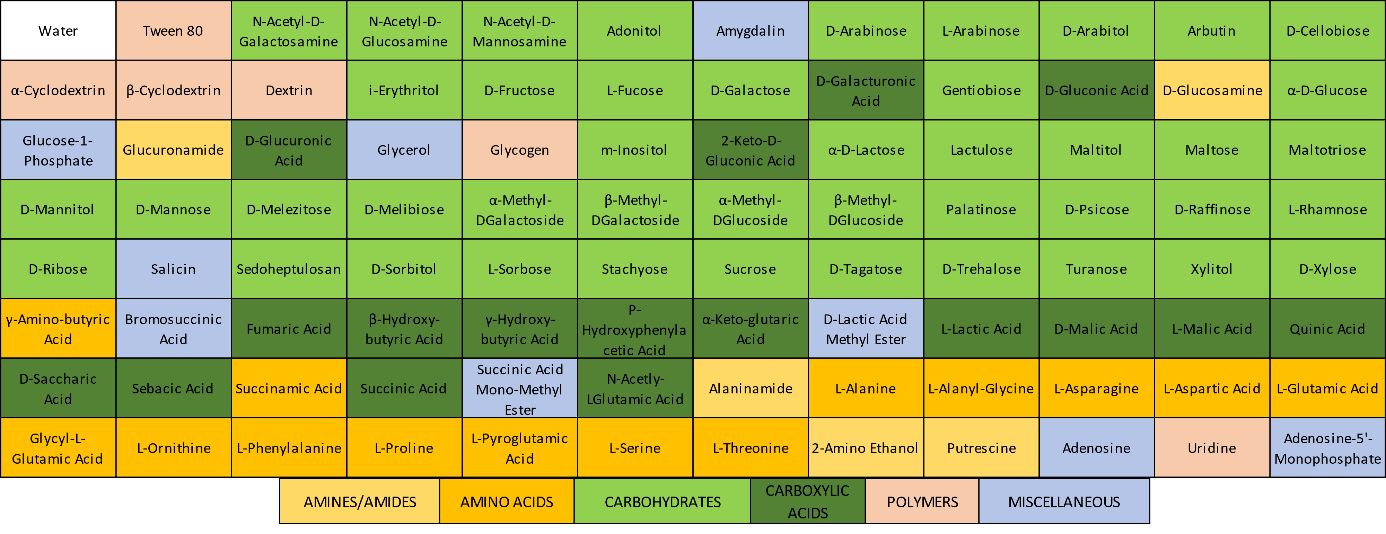


**Figure S1.** List of 96 substances inside the FF microplate and their grouping.


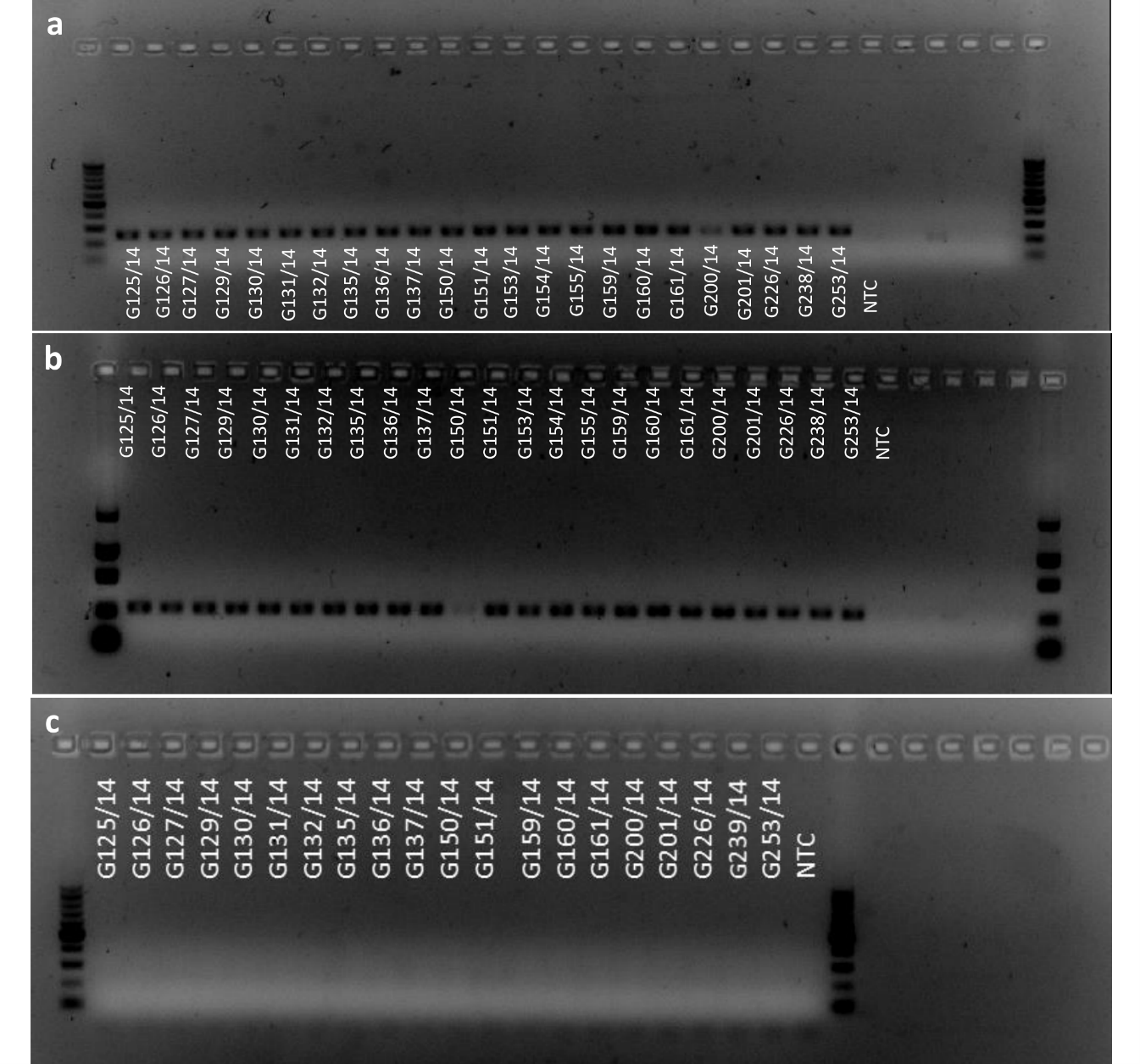


**Figure S2.** PCR amplifications using primers for: ITS1 (**a**) , β-tubulin *Neosartorya* spp. specific (**b**) and *A. fumigatus* specific (**c**).


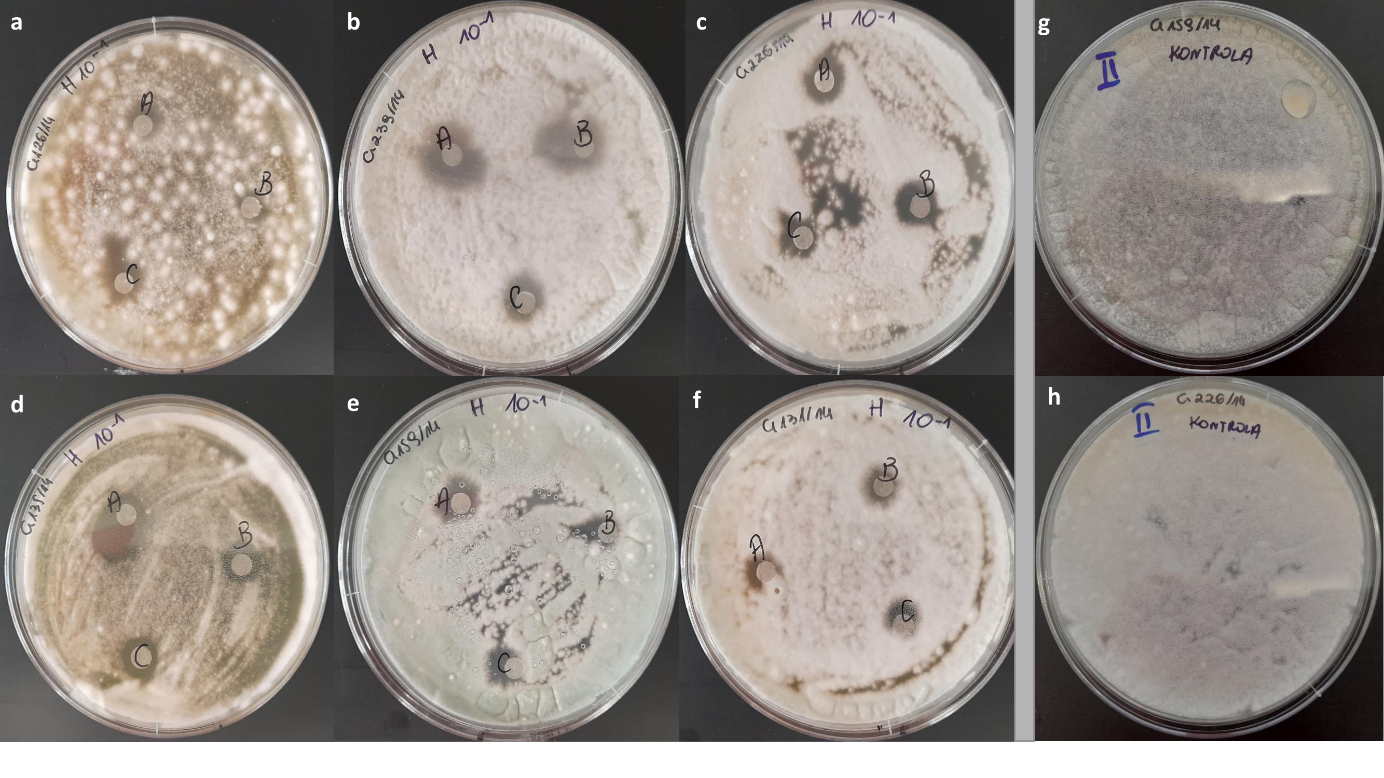


**Figure S3.** Inhibition zones in *Neosartorya* spp. growth formed under the influence of different dilutions of tea tree oil (**a**-**f**) and control plates (**g**-**h**).


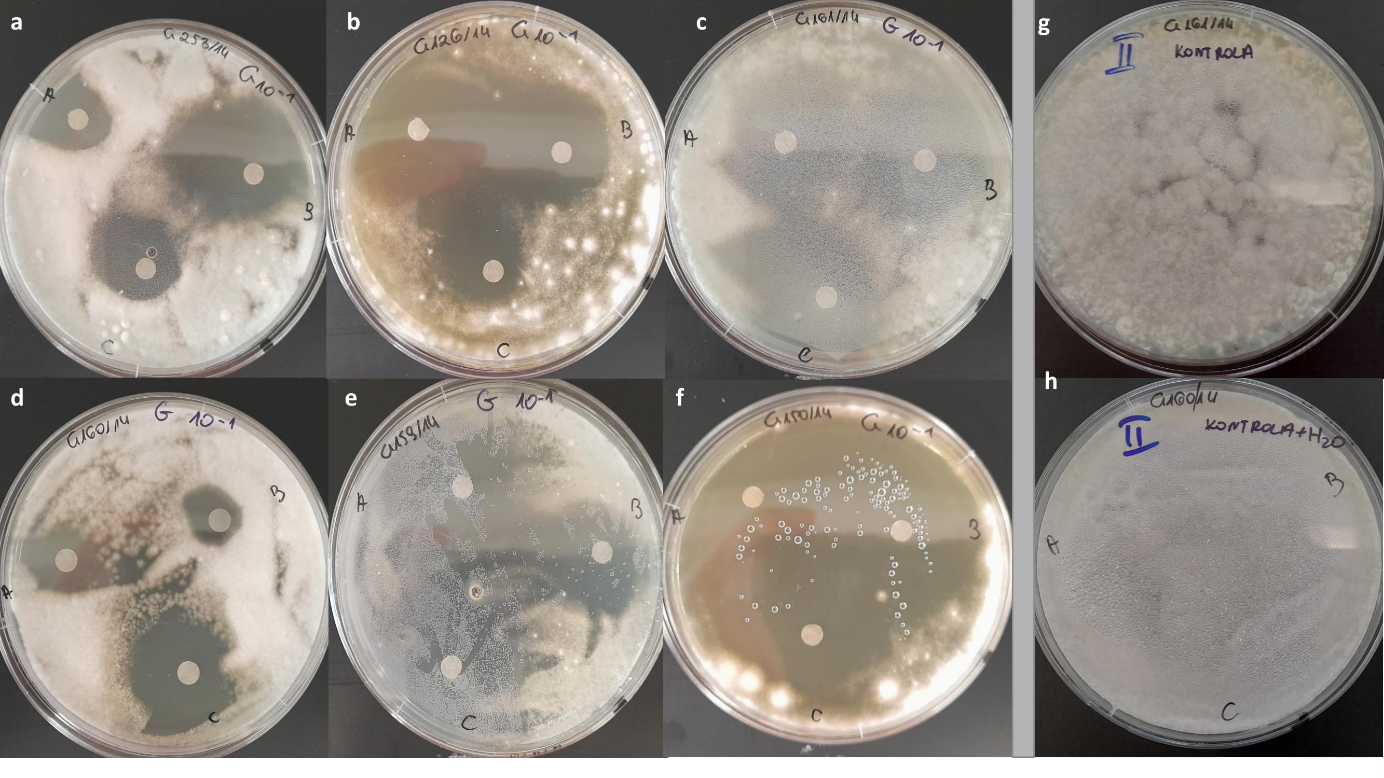


**Figure S4.** Inhibition zones in *Neosartorya* spp. growth formed under the influence of different dilutions of clove oil (**a**-**f**) and control plates (**g**-**h**).


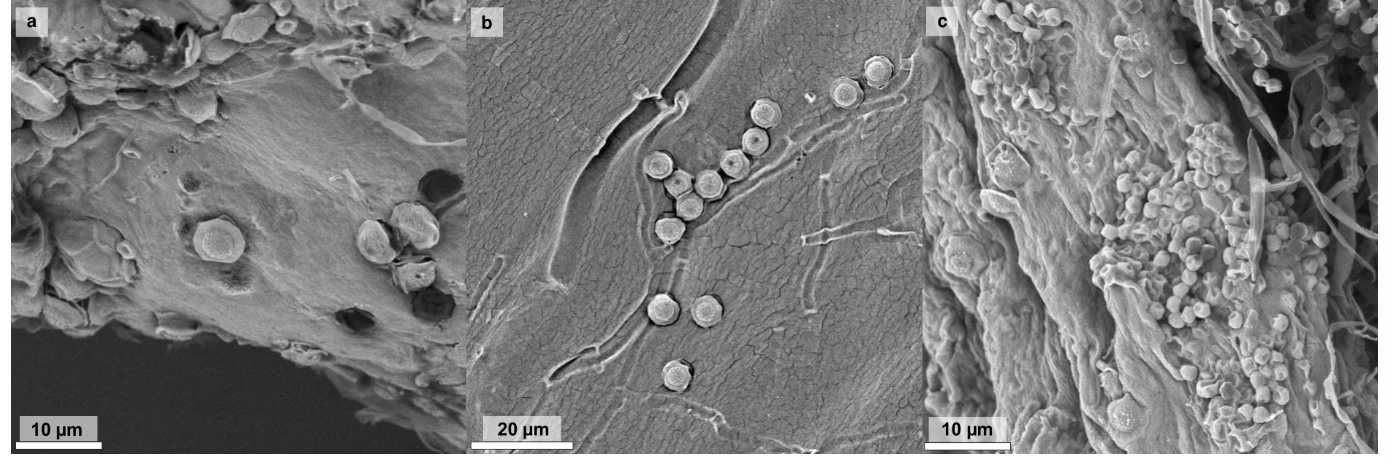


**Figure S5.** *Neosartorya* spp. ascospores observation. SEM imaging of isolates G130/14 (5000X magnification) (**a**), G150/14 (3000X magnification) (**b**), G132/14 (5000X magnification) (**c**).

**Table S1**. Amplification conditions for ITS1 and β-tubulin PCRs.

|  | ITS1 | β-TUB *Neosartorya* spp. and *Aspergillus fumigatus* | β-TUB *Aspergillus fumigatus* specific |
| --- | --- | --- | --- |
| Initial activation | 95°C for 3 min | 95 °C for 3 min | 94 °C for 3 min |
| Denaturation | 95°C for 15 s | 95 °C for 1 min | 94 °C for 5 s |
| Annealing | 55°C for 30 s | 59 °C for 1 min | 59 °C for 5 s |
| Extension | 72°C for 40 s | 72 °C for 1 min | 72 °C for 10 s |
| Final extension | 72°C for 7 min | 72 °C for 10 min | 72 °C for 1 min |
| Number of cycles | 35 | 35 | 35 |

**Table S2.** Description of isolates used in this study and NCBI database accession numbers.

| Isolate | Sample origin | Type of Sequence | NCBI Accession No. |
| --- | --- | --- | --- |
| G125/14 | Strawberry fruit | ITS | OQ300418.1 |
|  |  | B-TUB | OR500229 |
| G126/14 | Strawberry fruit | ITS | OQ300422.1 |
|  |  | B-TUB | OR597661 |
| G127/14 | Strawberry fruit | ITS | OQ300429.1 |
|  |  | B-TUB | OR597660 |
| G129/14 | Strawberry fruit | ITS | OQ300428.1 |
|  |  | B-TUB | OR597662 |
| G130/14 | Strawberry fruit | ITS | OQ303998.1 |
|  |  | B-TUB | OR597659 |
| G131/14 | Strawberry fruit | ITS | OQ303995.1 |
|  |  | B-TUB | OR597658 |
| G132/14 | Strawberry fruit | ITS | OQ300430.1 |
|  |  | B-TUB | OR597657 |
| G135/14 | Strawberry fruit | ITS | OQ300432.1 |
|  |  | B-TUB | OR608355 |
| G150/14 | Soil | ITS | OQ423135.1 |
|  |  | B-TUB | OR608366 |
| G151/14 | Soil | ITS | OQ306552.1 |
|  |  | B-TUB | OR608362 |
| G153/14 | Soil | ITS | OQ448879.1 |
|  |  | B-TUB | OR608354 |
| G154/14 | Soil | ITS | OQ306551.1 |
|  |  | B-TUB | OR608361 |
| G155/14 | Soil | ITS | OQ306553.1 |
|  |  | B-TUB | OR608356 |
| G159/14 | Soil | ITS | OQ306554.1 |
|  |  | B-TUB | OR608357 |
| G160/14 | Soil | ITS | OQ306555.1 |
|  |  | B-TUB | OR608359 |
| G161/14 | Soil | ITS | OQ448880.1 |
|  |  | B-TUB | OR608363 |
| G226/14 | Strawberry fruit | ITS | OQ306560.1 |
|  |  | B-TUB | OR608360 |
| G239/14 | Strawberry fruit | ITS | OQ306558.1 |
|  |  | B-TUB | OR608358 |
| G253/14 | Strawberry fruit | ITS | OQ306559.1 |
|  |  | B-TUB | OR608364 |
| G259/14 | Strawberry fruit | ITS | OQ306561.1 |
|  |  | B-TUB | OR608365 |

| **Isolate No.** | **Square position** | **Ascospores count in inoculating fluid with 75% transmittance [10^4^]** | | | | | | | **Average ascospores count [10^4^]** |
| --- | --- | --- | --- | --- | --- | --- | --- | --- | --- |
| **G150/14** | Top | 6 | 3 | 3 | 2 | 5 | 4 | 3 | 3.65 |
|  | Bottom | 3 | 4 | 2 | 5 | 4 | 4 | 3 |  |
| **G127/14** | Top | 5 | 7 | 5 | 6 | 2 | 4 | 4 | 4.79 |
|  | Bottom | 5 | 4 | 5 | 3 | 6 | 4 | 7 |  |
| **G132/14** | Top | 8 | 4 | 5 | 9 | 4 | 3 | 3 | 4.50 |
|  | Bottom | 5 | 3 | 4 | 3 | 7 | 3 | 2 |  |
| **G130/14** | Top | 3 | 5 | 4 | 3 | 2 | 3 | 2 | 3.65 |
|  | Bottom | 2 | 5 | 7 | 2 | 4 | 6 | 3 |  |
| **G135/14** | Top | 7 | 4 | 6 | 5 | 7 | 8 | 4 | 5.58 |
|  | Bottom | 5 | 6 | 3 | 8 | 8 | 4 | 3 |  |
| **Average ascospores counts from all tested *Neosartorya* spp. isolates** | | | | | | | | | **4.44** |

**Table S3.** Quantitative analysis of *Neosartorya* spp. isolates ascospores counts using Thoma cell counting chamber.
